# Non-viral, specifically targeted CAR-T cells achieve high safety and efficacy in B-NHL

**DOI:** 10.1038/s41586-022-05140-y

**Published:** 2022-08-31

**Authors:** Jiqin Zhang, Yongxian Hu, Jiaxuan Yang, Wei Li, Mingming Zhang, Qingcan Wang, Linjie Zhang, Guoqing Wei, Yue Tian, Kui Zhao, Ang Chen, Binghe Tan, Jiazhen Cui, Deqi Li, Yi Li, Yalei Qi, Dongrui Wang, Yuxuan Wu, Dali Li, Bing Du, Mingyao Liu, He Huang

**Affiliations:** 1 Shanghai Frontiers Science Center of Genome Editing and Cell Therapy, Shanghai Key Laboratory of Regulatory Biology, Institute of Biomedical Sciences and School of Life Sciences, East China Normal University, Shanghai, China; 2 Bone Marrow Transplantation Center, the First Affiliated Hospital, Zhejiang University School of Medicine, Hangzhou, China; 3 Liangzhu Laboratory, Zhejiang University Medical Center, Hangzhou, China; 4 Institute of Hematology, Zhejiang University, Hangzhou, China; 5 Zhejiang Province Engineering Laboratory for Stem Cell and Immunity Therapy, Hangzhou, China; 6 BRL Medicine, Inc., Shanghai, China; 7 PETCT Center, The First Affiliated Hospital, School of Medicine, Zhejiang University, Hangzhou, China

**Keywords:** B-cell lymphoma, Cell therapies, CRISPR-Cas9 genome editing

## Abstract

Recently, chimeric antigen receptor (CAR)-T cell therapy has shown great promise in treating haematological malignancies^1–7^. However, CAR-T cell therapy currently has several limitations^8–12^. Here we successfully developed a two-in-one approach to generate non-viral, gene-specific targeted CAR-T cells through CRISPR–Cas9. Using the optimized protocol, we demonstrated feasibility in a preclinical study by inserting an anti-CD19 CAR cassette into the *AAVS1* safe-harbour locus. Furthermore, an innovative type of anti-CD19 CAR-T cell with *PD1* integration was developed and showed superior ability to eradicate tumour cells in xenograft models. In adoptive therapy for relapsed/refractory aggressive B cell non-Hodgkin lymphoma (ClinicalTrials.gov, NCT04213469), we observed a high rate (87.5%) of complete remission and durable responses without serious adverse events in eight patients. Notably, these enhanced CAR-T cells were effective even at a low infusion dose and with a low percentage of CAR^+^ cells. Single-cell analysis showed that the electroporation method resulted in a high percentage of memory T cells in infusion products, and PD1 interference enhanced anti-tumour immune functions, further validating the advantages of non-viral, *PD1*-integrated CAR-T cells. Collectively, our results demonstrate the high safety and efficacy of non-viral, gene-specific integrated CAR-T cells, thus providing an innovative technology for CAR-T cell therapy.

## Abstract (web summary)

Non-viral CAR-T cells with gene-specific targeted integration are safe and effective in patients with lymphoma.

## Main

In recent years, chimeric antigen receptor (CAR)-T cell therapy has rapidly developed and it shows great potential in cancer therapy^1–7^. Nevertheless, some limitations still remain, including the complicated manufacturing process, high production cost, long preparation time and potential safety concerns of current therapies. The use of virus in CAR-T cell production is one area of concern, as the disadvantages of this approach include an increased risk of tumour development resulting from insertional mutagenesis^8,9^. Furthermore, specific responses to virus-derived DNA tend to impede CAR expression^10^,^11^, and virus manufacture frequently incurs high costs^12^. Although some strategies, such as using transposon systems^13–16^ and mRNA transduction^17–19^, are being exploited to generate CAR-T cells without virus, the low homogeneity of the final products caused by random integration and discontinued CAR expression become additional problems. Recently, several studies have shown that genome editing technologies can be applied to generate locus-specific integrated CAR-T cells by using an adeno-associated virus (AAV) vector as a template^20–22^. Furthermore, one preferential non-viral strategy was proposed to produce T cell products with point mutation correction and precise insertion of the T cell receptor (TCR) element^23^. Thus, to simultaneously solve the disadvantages of virus usage and random integration, here we developed non-viral, gene-specific targeted CAR-T cells through CRISPR–Cas9 and demonstrated their high safety and effectiveness in treating patients with relapsed/refractory B cell non-Hodgkin lymphoma (r/r B-NHL).

## Characteristics of AAVS1-19bbz cells

**Fig. 1:**
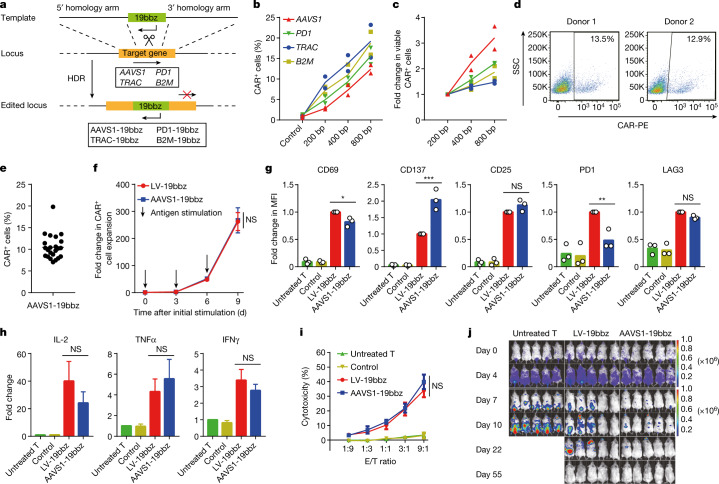
Non-viral, *AAVS1*-integrated CAR-T cells effectively eliminate tumour cells. **a**, Specific integration of the CAR cassette into the target locus by homologous recombination through CRISPR–Cas9. **b**,**c**, Percentage of CAR^+^ cells (**b**) and number of viable CAR^+^ cells (**c**) detected 7 d after electroporation using equimolar amounts of DNA templates with different homology arm lengths (*n* = 2 independent healthy donors). **d**, CAR expression in cells from two representative healthy donors determined 7 d after electroporation. SSC, side scatter. K, ×1,000. **e**, Percentage of CAR^+^ cells detected 7 d after electroporation (*n* = 23 independent healthy donors). **f**, Expansion of CAR^+^ cells after repeated stimulation with Raji cells. Data are shown as the mean ± s.e.m. (*n* = 3 independent healthy donors). **g**, Median fluorescence intensity (MFI) of CD69, CD137, CD25, PD1 and LAG3 expression in T cells detected after 24 h of co-culture with Raji cells (*n* = 3 independent healthy donors). CD3^+^ (untreated T, control) or CD3^+^CAR^+^ (LV-19bbz, AAVS1-19bbz) gated cells were analysed. **h**, Cytokine secretion measured by bead-based immunoassay in the supernatant after co-culture with Raji cells for 24 h. Data are shown as the mean ± s.e.m. (*n* = 3 independent healthy donors). IL-2, interleukin-2; TNFα, tumour necrosis factor α; IFNγ, interferon-γ. **i**, In vitro cytotoxicity against Raji cells as determined by lactate dehydrogenase (LDH) assay. E/T ratio, effector/target ratio. Data are shown as the mean ± s.e.m. (*n* = 3 independent healthy donors). **j**, Bioluminescence imaging of tumour cell growth following different treatments on the indicated days after CAR-T cell infusion (*n* = 5). The radiance scale (p s^–1^ cm^–^^2^ sr^–1^) is shown. Immunodeficient mice were injected intravenously with 2 × 10^5^ firefly luciferase (ffLuc)-transduced Raji cells, and 2 × 10^6^ CAR-T cells were administered intravenously after 5 d. Control samples were electroporated the same as AAVS1-19bbz cells except without single guide RNA (sgRNA) addition. The mean value is shown in **b**,**c**,**e**,**g**. *P* values were calculated by one-way ANOVA with Tukey’s multiple-comparisons test (**g**,**h**) or two-way ANOVA with Sidak’s multiple-comparisons test (**f**) or Tukey’s multiple-comparisons test (**i**). ***P   < 0.001, **P  <  0.01; NS, not significant. Source data

**Extended Data Fig. 1:**
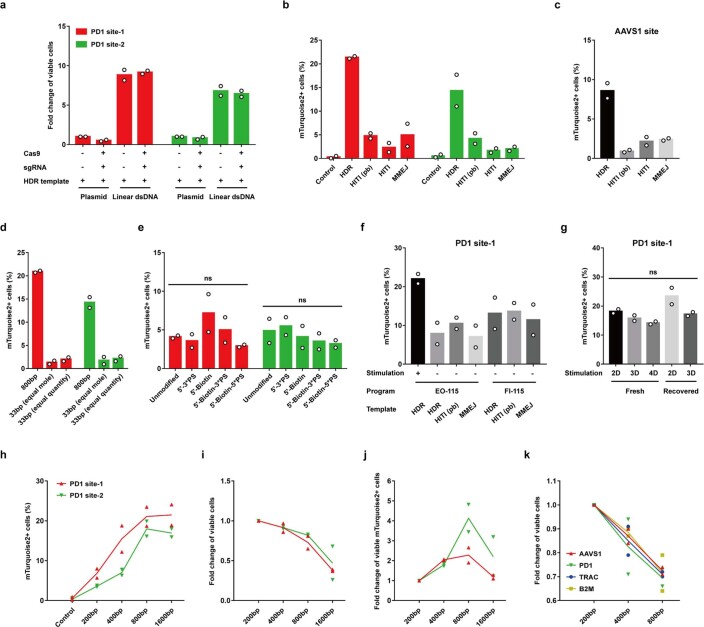
Optimization of the conditions for producing non-viral gene-specific targeted T cells. a-j, The sequence of the fluorescent protein mTurquoise2 was used as a target to optimize the conditions for generating non-viral gene-specific integrated T cells. a, Number of viable cells calculated 7 days after electroporation by using different protocols. Equal quantities of circular plasmid DNA and linear double-stranded DNA (dsDNA) were used. Due to acquisition of higher cell viability, templates in the form of linear dsDNA were chosen for all the following experiments. b-c, Homologous recombination efficiency of mTurquoise2 at two *PD1* sites (b) and one *AAVS1* site (c) by using different DNA templates. HDR, homology directed repair. HITI, homology-independent targeted integration. HITI (pb), HITI template with 50bp protection base pairs flanking the target sequence. MMEJ, microhomology-mediated end joining. d, Homologous recombination efficiency of mTurquoise2 using 33bp or 800bp homology arms. Equal mole or quantity of template harboring 33bp homology arms was used, compared with template with 800bp homology arms. e, Homologous recombination efficiency of mTurquoise2 by using unmodified or modified DNA templates with 200bp homology arms. PS, phosphorothioate. Biotin was modified at the first base pair from the 5’ side. PS was modified at the first three or five base pairs from the 5’ side. f, Homologous recombination efficiency of mTurquoise2 in unstimulated or stimulated T cells using different electroporation programs and DNA templates. g, Homologous recombination efficiency of mTurquoise2 in fresh or recovered T cells after stimulation for indicated days by using HDR templates with 800bp homology arms. h-j, Homologous recombination efficiency (h) and numbers of all viable cells (i) and viable mTurquoise2+ cells (j) were detected using mTurquoise2 templates with different homology arm lengths. k, Number of all viable cells was enumerated using CAR templates with different homology arm lengths. 800bp and 20bp homology arms were used in HDR and MMEJ templates, respectively (a–c, f). Equal moles of DNA template were used in b, c, e–k. The homologous recombination efficiency was determined 7 days after electroporation in b-k. All the experiments were performed in cells from two independent healthy donors. Mean value is shown in all the figures. P values were calculated by one-way ANOVA with Tukey’s multiple comparisons test (g) or two-way ANOVA with Tukey’s multiple comparisons test (e). Source data

**Extended Data Fig. 2:**
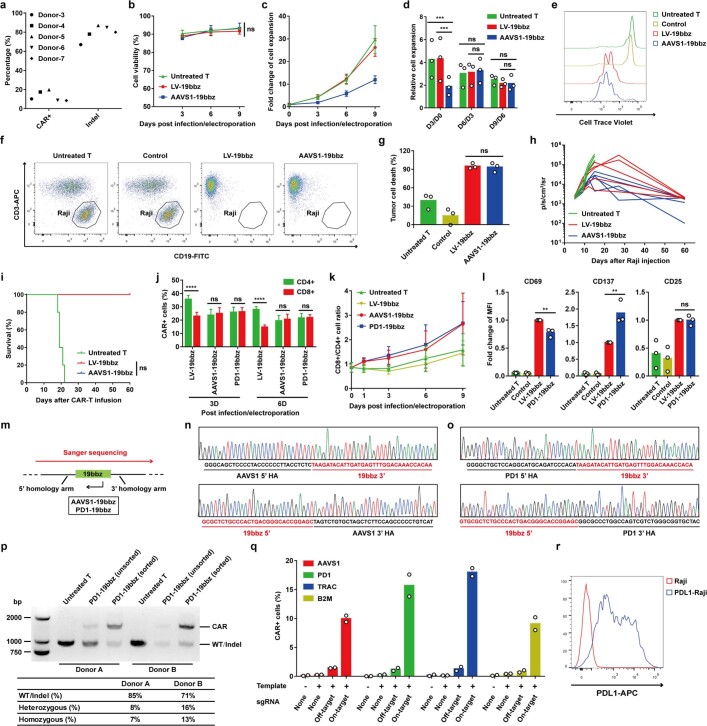
Comparison of non-viral gene-specific integrated CAR-T cells and lentivirus-produced CAR-T cells. a, Percentages of CAR integration and *AAVS1* indels in total T cells were detected 7 days after electroporation in five representative healthy donors. b, Cell viability detected by trypan blue staining on indicated days post infection/electroporation. Data are mean ± SEM (n = 3 independent healthy donors). c-d, Absolute (c) and relative (d) rates of T cell growth in vitro (n = 3 independent healthy donors). Data are mean ± SEM in c. e, Representative histogram showing Cell Trace Violet staining of T cells after co-culture with mitomycin C-treated Raji cells for 5 days. This experiment was repeated at least three times using different donors. f-g, In vitro cytotoxicity against Raji cells determined by flow cytometry. f, Representative plots showing lysis of Raji cells following 18 h co-culture. g, The percentage of Raji tumor cell death detected by flow cytometry-based cytotoxicity assay (n = 3 independent healthy donors). h, Bioluminescence kinetics of tumor cell growth following different treatments (n = 5). Immunodeficient mice were injected intravenously with 2×10^5^ ffLuc-transduced Raji cells and 2×10^6^ CAR-T cells were administered intravenously after 5 days. i, Kaplan-Meier analysis of survival of mice in h. j, Percentages of CAR+ cells in CD4+ and CD8+ cells determined 3 or 6 days after infection/electroporation. Data are mean ± SEM (n = 4 independent healthy donors). k, Ratio of CD4+ and CD8+ cells on indicated days post infection/electroporation. Data are mean ± SEM (n = 3 independent healthy donors). l, MFI of CD69, CD137 and CD25 expression in T cells detected by flow cytometry after 24 h co-culture with PD-L1 expressing Raji cells (n = 3 independent healthy donors). m, For the samples of AAVS1-19bbz and PD1-19bbz, CAR+ cells were sorted by fluorescence-activated cell sorting (FACS). Genomic DNA was used as template to amplify PCR products across the homology arms. Sanger sequencing was performed from end to end, outside of homology arms. n-o, Sequences of 5’ and 3’ junction sites between the homology arm and CAR cassette at the *AAVS1* (n) and *PD1* (o) locus. p, Genotyping of PD1-19bbz cells in two independent healthy donors by calculating the intensity ratio of WT/indel and CAR specific bands in unsorted and sorted CAR+ cells. WT, wild type. q, Non-specific integration of CAR elements was tested 7 days after electroporation by using recombinant spCas9 and different combinations of DNA template and sgRNA (n = 2 independent healthy donors). For the groups of AAVS1, PD1 and TRAC templates, one B2M sgRNA with high cleavage efficiency was used as off-target sgRNA. For the B2M template group, one TRAC sgRNA with high cleavage efficiency was used as off-target sgRNA. The off-target groups were designed to detect non-targeted integration under a hypothesized condition that sgRNA had very high off-target cleavage efficiency. r, PD-L1 expression in Raji cell lines. Control samples were electroporated the same as AAVS1-19bbz or PD1-19bbz cells except without sgRNA addition. CD3+ (Untreated T, Control) or CD3+/CAR+ (LV-19bbz, AAVS1-19bbz, PD1-19bbz) gated cells were analysed in e, l. Mean value is shown in d, g, l, q. P values were calculated by one-way ANOVA with Tukey’s multiple comparisons test (g, l), two-way ANOVA with Tukey’s multiple comparisons test (b, d) and Sidak’s multiple comparisons test (j) or log-rank Mantel-Cox test (i). Source data

First, we sought to optimize the protocol for producing non-viral, gene-specific integrated T cells. A homology-directed repair (HDR) template, in the form of linear double-stranded DNA (dsDNA), was found to achieve high homologous recombination efficiency and cell viability (Fig. 1a and Extended Data Fig. 1a–c). More viable cells carrying a targeted gene integration were acquired when electroporation was carried out in stimulated T cells by applying 800-bp homology arms (Fig. 1b,c and Extended Data Fig. 1d–k). After confirmation of an optimal protocol, for proof of concept, we first chose to target the CAR-expressing cassette to the *AAVS1* safe harbour to evaluate whether this approach would affect the properties of the CAR-T cells. An anti-CD19 CAR sequence containing 4-1BB and CD3ζ (named 19bbz) was constructed. The integration efficiency of 19bbz into *AAVS1* was about 10% (up to 19.8%), and the indel percentage ranged from 67% to 87% (Fig. 1d,e and Extended Data Fig. 2a,m,n,q). Next, we comprehensively compared *AAVS1*-integrated (AAVS1-19bbz) and lentivirus-produced (LV-19bbz) anti-CD19 CAR-T cells. Although the electroporation procedure itself led to some cell damage, T cell expansion was not impaired and high cell viability was detected after thorough recovery (Extended Data Fig. 2b–d). While lentivirus infection resulted in a higher percentage of CAR^+^ cells among CD4^+^ cells than among CD8^+^ cells, integration was unbiased between CD4^+^ and CD8^+^ cells by the electroporation strategy (Extended Data Fig. 2j). Notably, electroporation increased the ratio of CD8^+^ to CD4^+^ T cells when compared with lentiviral transduction (Extended Data Figs. 2k and 8d–f), which was consistent with a previous study^23^. We observed that AAVS1-19bbz cells responded to tumour cells as LV-19bbz cells did (Fig. 1f–h and Extended Data Fig. 2e). By contrast, some differences were found in cell marker expression and cytokine secretion. Notably, like LV-19bbz cells, AAVS1-19bbz cells vigorously eradicated tumour cells in vitro and in vivo (Fig. 1i,j and Extended Data Fig. 2f–i). Taken together, these results demonstrate that the strategy to produce non-viral, gene-specific targeted CAR-T cells is feasible.

## PD1-19bbz cells outperform LV-19bbz cells

**Fig. 2:**
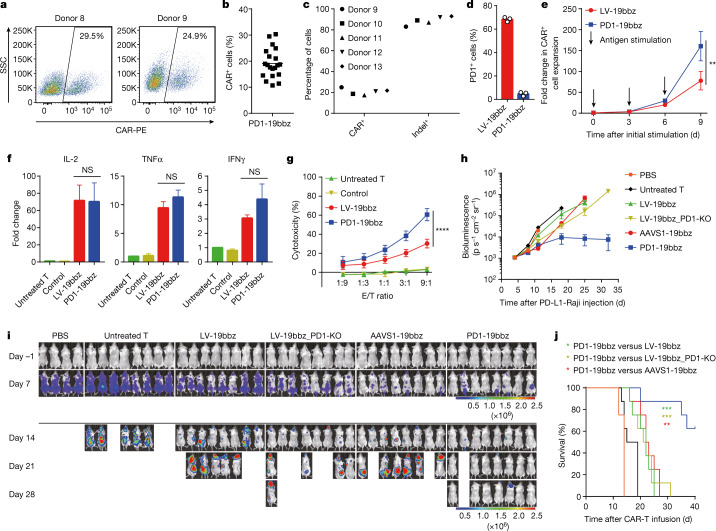
Non-viral, *PD1*-integrated CAR-T cells outperform lentivirus-produced CAR-T cells. **a**, CAR expression in cells from two representative healthy donors determined 7 d after electroporation. **b**, Percentage of CAR^+^ cells detected 7 d after electroporation (*n* = 20 independent healthy donors). **c**, Percentages of CAR integration and *PD1* indels in total T cells detected 7 d after electroporation in five representative healthy donors. **d**, Percentage of cells with PD1 expression detected by flow cytometry in CD3^+^CAR^+^ gated cells after 24 h of co-culture with PD-L1-expressing Raji cells (*n* = 3 independent healthy donors). **e**, Expansion of CAR^+^ cells after repeated stimulation with PD-L1-expressing Raji cells. Data are shown as the mean ± s.e.m. (*n* = 3 independent healthy donors). **f**, Cytokine secretion measured by bead-based immunoassay in the supernatant after co-culture with PD-L1-expressing Raji cells for 24 h. Data are shown as the mean ± s.e.m. (*n* = 3 independent healthy donors). **g**, In vitro cytotoxicity against PD-L1-expressing Raji cells determined by LDH assay. Data are shown as the mean ± s.e.m. (*n* = 3 independent healthy donors). **h**–**j**, Immunodeficient mice were injected intravenously with 5 × 10^5^ ffLuc-transduced PD-L1-expressing Raji cells, and 1 × 10^6^ CAR-T cells were administered intravenously after 5 d. **h**,**i**, Bioluminescence kinetics (**h**) and imaging (**i**) of tumour cell growth following different treatments (*n* = 4 or 8). Data are shown as the mean ± s.e.m. in **h**. Imaging on the indicated days after CAR-T cell infusion and the radiance scale (p s^–1^ cm^–^^2^ sr^–1^) are shown in **i**. **j**, Kaplan–Meier analysis of survival of the mice in **i**. Control samples were electroporated the same as PD1-19bbz cells except without sgRNA addition. The mean value is shown in **b**,**d**. *P* values were calculated by one-way ANOVA with Tukey’s multiple-comparisons test (**f**), two-way ANOVA with Sidak’s multiple-comparisons test (**e**) or Tukey’s multiple-comparisons test (**g**), or a log-rank Mantel–Cox test (**j**). ****P  <  0.0001, **P  <  0.01; NS, not significant. Source data

**Extended Data Fig. 3:**
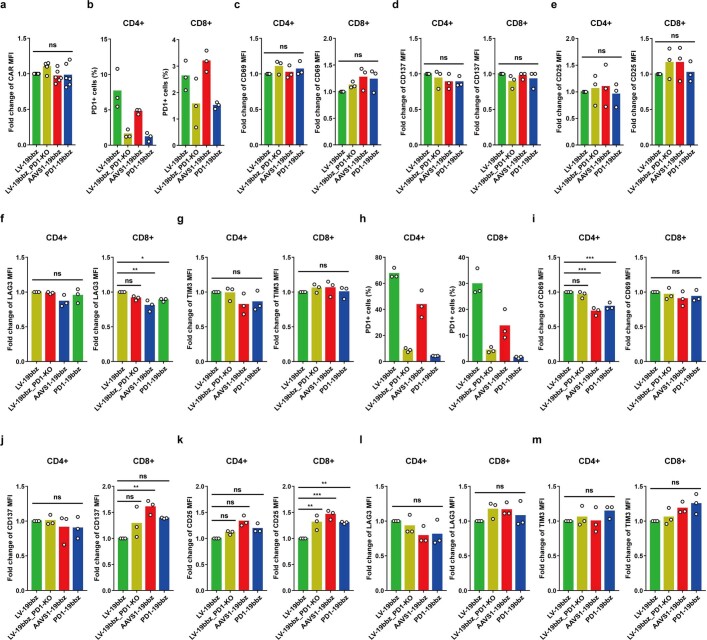
Non-viral gene-specific integrated CAR-T cells exhibit similar antigen-independent and -dependent tonic signalling as lentivirus-produced CAR-T cells. a, MFI of CAR expression in T cells detected by flow cytometry without antigen stimulation (n = 6 independent healthy donors). b, Percentages of PD1 expression in CD4+/CAR+ and CD8+/CAR+ cells detected by flow cytometry without antigen stimulation. c-g, MFI of CD69, CD137, CD25, LAG3 and TIM3 expression in CD4+/CAR+ and CD8+/CAR+ cells without antigen stimulation. h, Percentages of PD1 expression in CD4+/CAR+ and CD8+/CAR+ cells after 24 h co-culture with Raji cells. i-m, MFI of CD69, CD137, CD25, LAG3 and TIM3 expression in CD4+/CAR+ and CD8+/CAR+ cells after 24 h co-culture with Raji cells. The experiments were performed in three independent healthy donors (b-m). Mean value is shown in all the figures. P values were calculated by one-way ANOVA with Tukey’s multiple comparisons test (a, c-g, i-m). Source data

**Extended Data Fig. 4:**
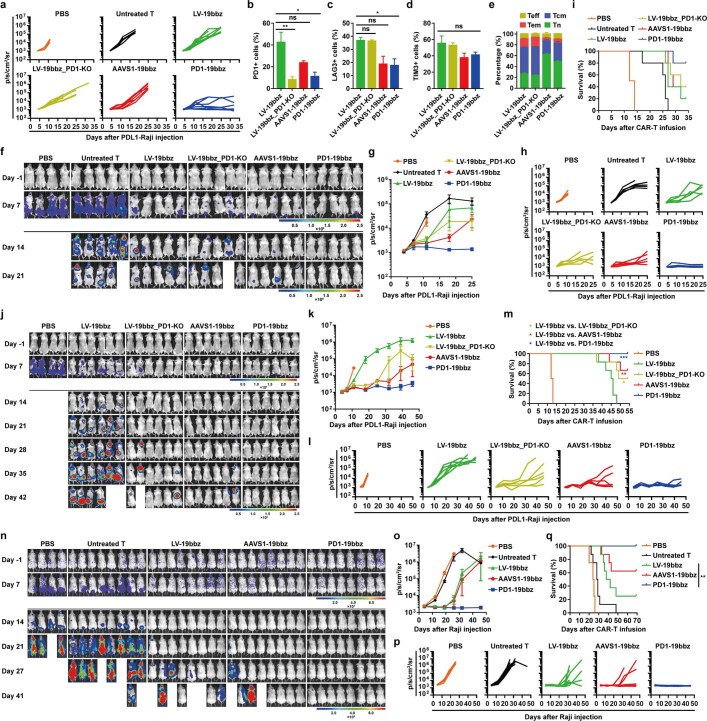
Non-viral *PD1*-integrated CAR-T cells effectively eradicate tumor cells with either high or low PD-L1 expression at a low or high infusion dose. a-e, Immunodeficient mice were injected intravenously with 5 × 10^5^ ffLuc-transduced PD-L1 expressing Raji cells and 1×10^6^ CAR-T cells were administered intravenously after 5 days. a, Bioluminescence kinetics of tumor cell growth in each treatment group (n = 4 or 8). b-d, Percentages of PD1, LAG3 and TIM3 expression in peripheral CAR-T cells detected 7 days after CAR-T cell infusion. Data are mean ± SEM (n = 3). e, After CAR-T cell infusion for 7 days, the T cell subset differentiation in peripheral CAR-T cells was analysed according to CD45RO/CD62L expression by flow cytometry. Data are mean ± SEM (n=3). f-i, Immunodeficient mice were injected intravenously with 5×10^5^ ffLuc-transduced PD-L1 expressing Raji cells and 2 × 10^6^ CAR-T cells were administered intravenously after 5 days. The T cells were harvested from the same healthy donor as that in Fig. 2h–j and a–e. f-h, Bioluminescence imaging (f) and kinetics (g, h) of tumor cell growth following different treatments (n = 4 or 5). Data are mean ± SEM in g. i, Kaplan-Meier analysis of survival of mice in f. j-m, Immunodeficient mice were injected intravenously with 5×10^5^ ffLuc-transduced PD-L1 expressing Raji cells and 2×10^6^ CAR-T cells were administered intravenously after 5 days. The T cells were harvested from one healthy donor who was different from that in Fig. 2h-j and a–i. j-l, Bioluminescence imaging (j) and kinetics (k, l) of tumor cell growth following different treatments (n = 4 or 6). Data are mean ± SEM in k. m, Kaplan-Meier analysis of survival of mice in j. n-q, Immunodeficient mice were injected intravenously with 2×10^5^ ffLuc-transduced Raji cells and 1×10^6^ CAR-T cells were administered intravenously after 5 days. n-p, Bioluminescence imaging (n) and kinetics (o, p) of tumor cell growth following different treatments (n = 4 or 8). Data are mean ± SEM in o. q, Kaplan-Meier analysis of survival of mice in n. The imaging on indicated days after CAR-T cell infusion and the radiance scale (p/s/cm^2^/sr) are shown (f, j, n). P values were calculated by one-way ANOVA with Tukey’s multiple comparisons test (b-d) or log-rank Mantel-Cox test (m, q). Source data

Given that blockage of the PD1–PD-L1 pathway has been reported to improve the anti-tumour activity of CAR-T cells^24–27^, we set out to develop an enhanced type of CAR-T cells by integrating an anti-CD19 CAR sequence into the *PD1* gene (PD1-19bbz) (Fig. 1a and Extended Data Fig. 2m,o–q). CAR expression was observed in about 20% (up to 30.3%) of healthy donor T cells, and a high indel percentage (83–93%) and PD1 impairment were detected (Fig. 2a–d). PD1-19bbz cells had higher proliferation than LV-19bbz cells after repeated stimulation with PD-L1-expressing Raji cells (Fig. 2e and Extended Data Fig. 2r). As indicated by other reports^28–30^, PD1 disruption did not affect the elevation of activation markers and cytokine secretion to counteract targeted tumour cells (Fig. 2f and Extended Data Fig. 2l). To fully understand the properties of PD1-19bbz cells, we manufactured CAR-T cells with *PD1* knockout using lentivirus and CRISPR–Cas9 (LV-19bbz_PD1-KO) and performed parallel assays in various groups. Although expression of some cell markers was different, PD1-19bbz cells in general exhibited similar CAR expression levels and antigen-independent and antigen-dependent tonic signalling as other CAR-T cells (Extended Data Fig. 3). Notably, in comparison with other groups, PD1-19bbz cells showed more robust clearance of tumour cells expressing either high or low levels of PD-L1 (Fig. 2g–j and Extended Data Fig. 4). Collectively, these data indicate that non-viral, *PD1*-integrated CAR-T cells have the potential to more effectively eliminate tumour cells.

## r/r B-NHL treated with PD1-19bbz cells

**Fig. 3:**
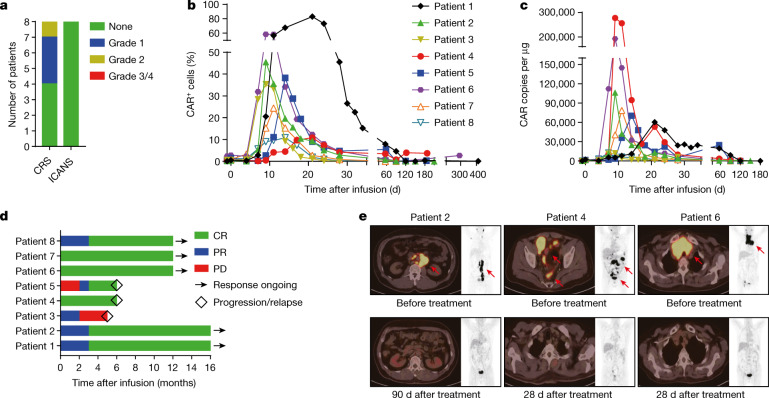
Non-viral, *PD1*-integrated CAR-T cells potently eliminate tumour cells in patients with r/r B-NHL without serious toxicity. **a**, Occurrence of CRS and ICANS after treatment. **b**, Percentage of CAR^+^ cells among the peripheral blood T cells of patients on the indicated days before and after infusion. **c**, CAR copy number in genomic DNA from the peripheral blood of patients on the indicated days before and after infusion. **d**, Treatment response and duration of response after infusion. PD, progressive disease. **e**, PET-CT scans of three representative patients before and after treatment. Red arrows indicate tumour lesions. Source data

**Extended Data Fig. 5:**
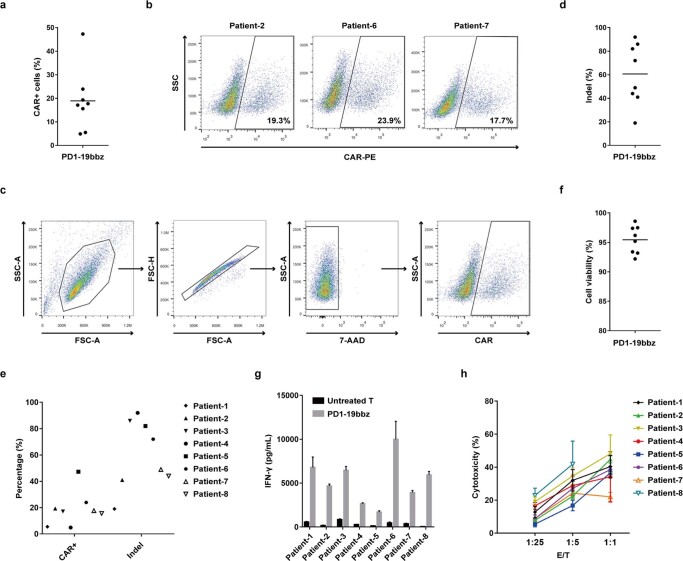
In vitro evaluation of non-viral *PD1*-targeted CAR-T cell products. a, Percentage of CAR+ cells in the final products of r/r B-NHL patients (n = 8 patient donors). b, CAR expression determined in three representative patient donors. c, Gating strategy for the detection of CAR expression. d-e, Percentages of CAR integration (e) and *PD1* indels (d, e) in the final products (n = 8 patient donors). f, Cell viability of the final products detected by trypan blue staining (n = 8 patient donors). g, IFN-γ secretion measured by ELISA in the supernatant after co-culture with Nalm-6 cells for 18–24 h. Data are mean ± SD (n = 3 technical replicates). h, In vitro cytotoxicity against Nalm-6 cells determined using LDH assay. E/T, effector/target. Data are mean ±SD (n = 3 technical replicates). Mean value is shown in a, d, f. Source data

**Extended Data Fig. 6:**
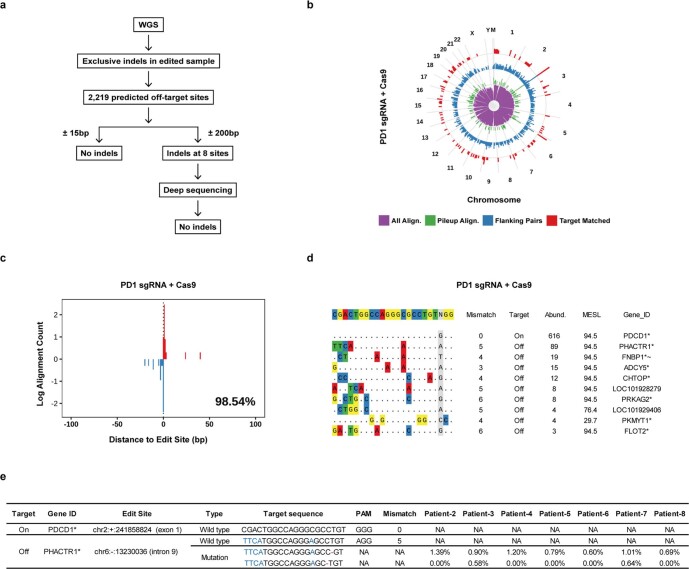
Off-target detection in non-viral *PD1*-integrated CAR-T cell products. a, Off-target detection by whole genome sequencing (WGS) and deep sequencing. The genomic DNA of untreated T cells and the infusion product of patient-2 was subjected to 100× WGS. A total of 2,219 potential off-target sites (not including those around the on-target site) were predicted by Cas-OFFinder and compared with exclusive indels in the edited sample by bioinformatics. No indel events were detected within 15bp upstream and downstream (±15bp) of the sites. Indels were found within 200bp upstream and downstream (±200bp) of eight sites. Deep sequencing (10,000× coverage) was then performed to validate these indel events. While no indels were detected at five sites, indels at the other three sites were variances of one unit length on nucleotide repeats and thus were not considered to be true off-target events. b-e, Identification of off-target sites by iGUIDE. b, Genomic distribution of oligonucleotide (dsODN) incorporation sites by bioinformatic characteristics. The ring indicates the human chromosomes aligned end-to-end, plus the mitochondrial chromosome (labeled M). The frequency of cleavage and subsequent dsODN incorporation is shown on a log scale on each ring (pooled over 10 Mb windows). The purple inner most ring plots all alignments identified. The green ring shows three or more unique alignments that overlap with each other (pileup alignment). The blue ring shows alignments that can be found on either side of a dsODN incorporation site (flanking pairs). The red ring shows reads with matches to the gRNA (allowing < 6 mismatches) within 100 bp (target matched). c, Distance distribution of identified incorporation sites from the *PD1* on-target locus. The percentage of incorporations within 100 bp of the on-target site is shown. d, Potential off-target cleavage sites identified by iGUIDE. Abund., the total number of unique alignments associated with the site. MESL, maximum edit site likelihood. Gene_ID, an identifier indicating the nearest gene. Symbols after the gene name indicate: * the site is within the transcription unit of the gene, ~ the gene appears on the cancer-association list. e, Deep sequencing (50,000× coverage) to validate off-target events at the *PHACTR1* site in different infusion products.

**Extended Data Fig. 7:**
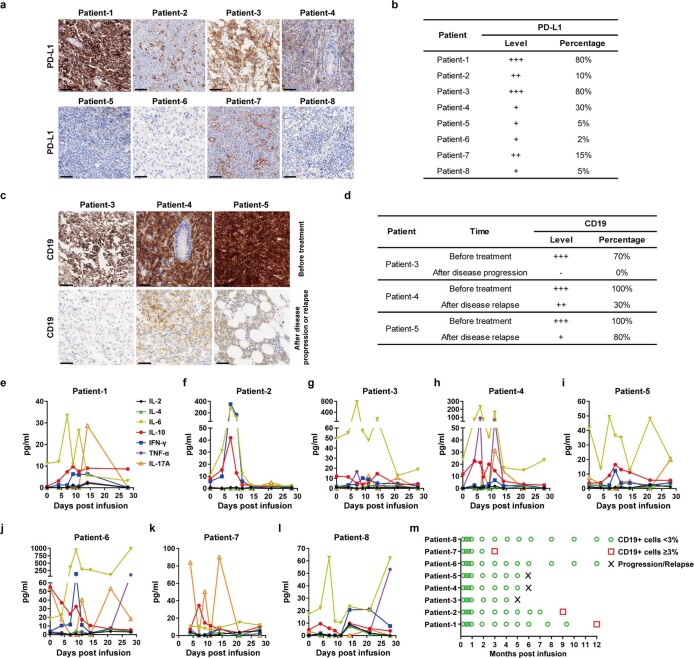
PD-L1 and CD19 expression in the tumor tissues, serum cytokine profiles and peripheral CD19+ cell percentage in the patients treated with non-viral *PD1*-targeted CAR-T cells. a. Representative immunohistochemistry (IHC) staining of PD-L1 protein expression in the patients’ tumor tissues before treatment. b. Evaluation of PD-L1 expression level and percentage in IHC staining. c, Representative IHC staining of CD19 protein expression in the patients’ tumor tissues before treatment and after disease progression or relapse. d, Evaluation of CD19 expression level and percentage in IHC staining. e-l, Serum cytokines including IL-2, IL-4, IL-6, IL-10, IFN-γ, TNF-α and IL-17A were assessed in eight r/r B-NHL patients on indicated days after infusion. m, Percentage of CD19+ cells in the peripheral lymphocytes of patients on indicated months after infusion. Scale bars represent 50 μm. Source data

On the basis of our preclinical experimental data, we then proceeded to carry out a phase 1 clinical trial to evaluate the safety and efficacy of PD1-19bbz cells in treating patients with r/r B-NHL (ClinicalTrials.gov, NCT04213469). Eight patients who had not previously been treated with CAR-T cell therapy were enrolled. In the final infusion products, the average percentages of CAR integration and *PD1* indels were about 20% and 60%, respectively (Extended Data Fig. 5a–e and Supplementary Table 1). The infusion products had a cell viability of greater than 90% and responded to and eradicated tumour target cells in vitro (Extended Data Fig. 5f–h). Low-frequency off-target events at one site in *PHACTR1* (phosphatase and actin regulator 1), identified by iGUIDE^31^, an improvement of the unbiased GUIDE-seq method^32^, were validated by deep sequencing (Extended Data Fig. 6 and Supplementary Tables 2–4 and 8). Depletion of PHACTR1 is not expected to bring about negative consequences because PHACTR1 is not reported to be expressed in T cells. Patients were given a lymphodepleting chemotherapy regimen using combined cyclophosphamide and fludarabine, followed by one infusion of PD1-19bbz cells with a dose of 0.56 × 10^6^ to 2.35 × 10^6^ cells per kg body weight (Extended Data Fig. 7a,b, Extended Data Table 1 and Supplementary Tables 5 and 6). While all patients experienced transient and reversible haematological toxicity events mainly related to the chemotherapy pretreatment, no other high-grade (≥3) adverse events (AEs) were found (Supplementary Table 7). Mild cytokine release syndrome (CRS) was observed in some patients, while immune effector cell-associated neurotoxicity syndrome (ICANS) did not occur (Fig. 3a and Extended Data Fig. 7e–l). PD1-19bbz cells proliferated and persisted in vivo (Fig. 3b,c and Extended Data Fig. 7m). During a median observation period of 12 months, complete remission (CR) was achieved in seven of the eight patients (87.5%), as shown by positron emission tomography and computed tomography (PET-CT) scan. Durable responses were found in five patients at the time of last follow-up, whereas disease relapse was detected in two patients at 6 months (Fig. 3d,e, Extended Data Fig. 7c,d and Extended Data Table 1). Partial remission (PR) was observed in the remaining patient (1/8); thus, the best objective response rate reached was 100% in all patients. Of note, PD1-19bbz cells were effective even at a low infusion dose and with a low percentage of CAR^+^ cells, thereby indicating the high potency of these *PD1*-integrated CAR-T cells. Together, these data demonstrate that non-viral, *PD1*-integrated CAR-T cells have high safety and efficacy for patients with r/r B-NHL.

## Single-cell analysis of PD1-19bbz cells

**Fig. 4:**
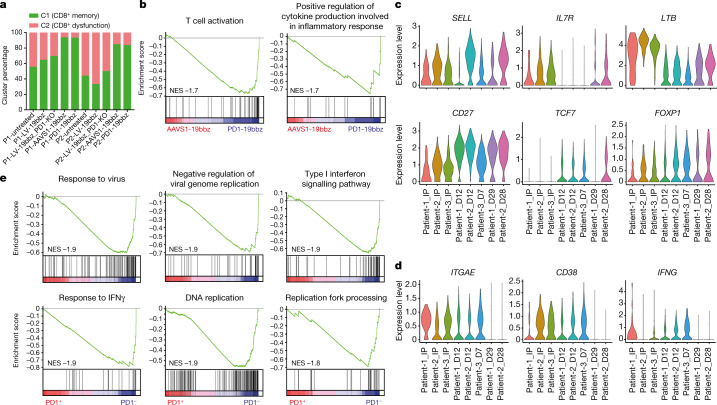
scRNA-seq analysis of non-viral, *PD1*-integrated CAR-T cells. **a**, Percentages of cluster 1 (C1) and cluster 2 (C2) in two donor samples prepared by different methods. C1 and C2 were generated by clustering cells on the basis of expression of CD8^+^ memory and dysfunction/cytotoxicity marker genes, respectively. P1, patient 1; P2, patient 2. **b**, GSEA of CD8^+^ T cells comparing AAVS1-19bbz and PD1-19bbz cells. Enriched gene sets in PD1-19bbz cells and the normalized enrichment score (NES) are shown. **c**,**d**, Violin plots showing the expression of memory (**c**) and dysfunction/cytotoxicity (**d**) genes in CD8^+^CAR^+^ cells from three patients before and after infusion. Data for the sample from patient 3 taken after 28 d of treatment are not shown owing to an unreliable low CAR^+^ cell number. D, days after infusion; IP, infusion product. **e**, GSEA comparing CD8^+^CAR^+^PD1^+^ and CD8^+^CAR^+^PD1^–^ cells from three patients after 7 or 12 d of infusion. The top six most enriched gene sets in CD8^+^CAR^+^PD1^–^ cells and the NES are shown. Source data

**Extended Data Fig. 8:**
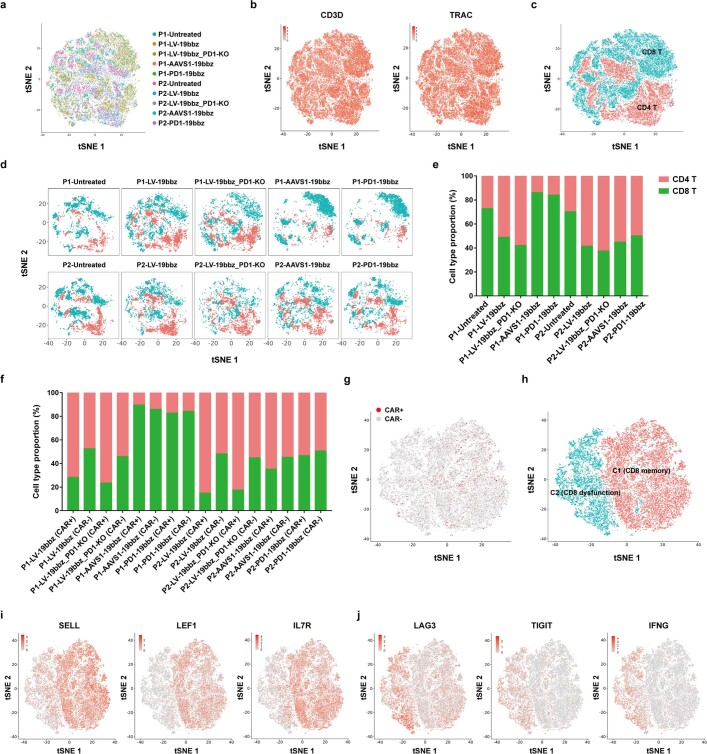
Single-cell RNA sequencing analysis of CAR-T cells prepared by different methods. a, Overview of the 63,789 cells that passed quality control (QC) for single-cell analysis. Cells are color coded by sample, respectively, in the t-distributed stochastic neighbor embedding (tSNE) plot. b, Expression of T cell marker genes (CD3D, TRAC) in the tSNE plots. c, Cells are color coded by CD4+ and CD8+ cells in the tSNE plot. d, tSNE plots showing CD4+ and CD8+ cell clusters in each sample. e-f, CD4+ and CD8+ cell proportion in total (e), CAR+ and CAR− (f) samples. g-j, CD8+ T cells were analysed in the samples prepared by different methods. g, Distribution of CAR+ and CAR− cells in the tSNE plot. h, tSNE plot showing two clusters in the samples. Cluster 1 (C1) and cluster 2 (C2) were generated by clustering CD8 memory and dysfunction/cytotoxicity marker genes, respectively. i, Expression of representative CD8 memory genes (SELL, LEF1, IL7R) in the tSNE plots. j, Expression of representative CD8 dysfunction/cytotoxicity genes (LAG3, TIGIT, IFNG) in the tSNE plots. Source data

**Extended Data Fig. 9:**
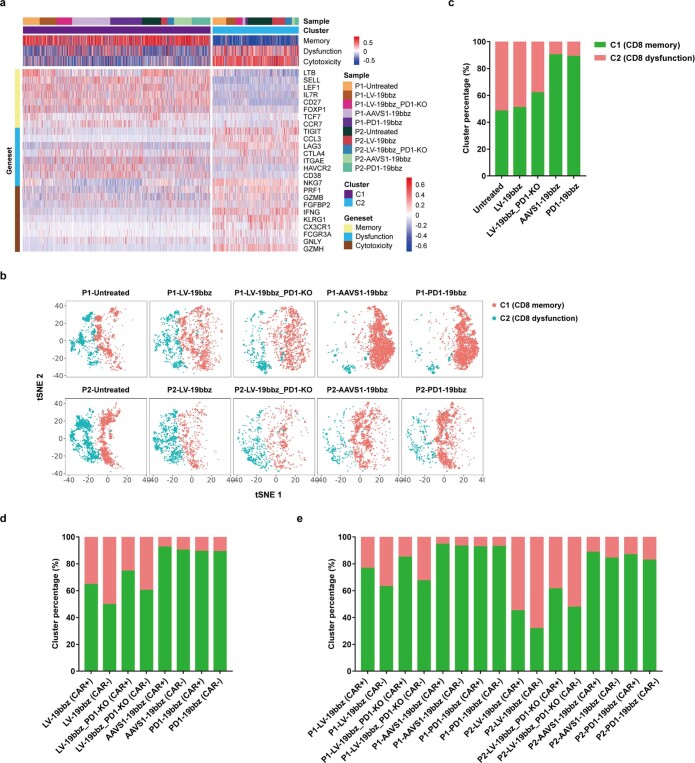
Proportion of CD8 memory and dysfunction clusters in CAR-T cells prepared by different methods. a, Heat map showing scaled expression of memory, dysfunction and cytotoxicity genes in two CD8+ T cell clusters. The gene set variation analysis (GSVA) scores of CD8 memory, dysfunction and cytotoxicity signatures are shown at the top. b, tSNE plots showing C1 and C2 in each sample. c, Percentages of C1 and C2 in mixed samples. d-e, Comparison of C1 and C2 proportion between CAR+ and CAR− cells in mixed (d) and individual (e) samples. Source data

**Extended Data Fig. 10:**
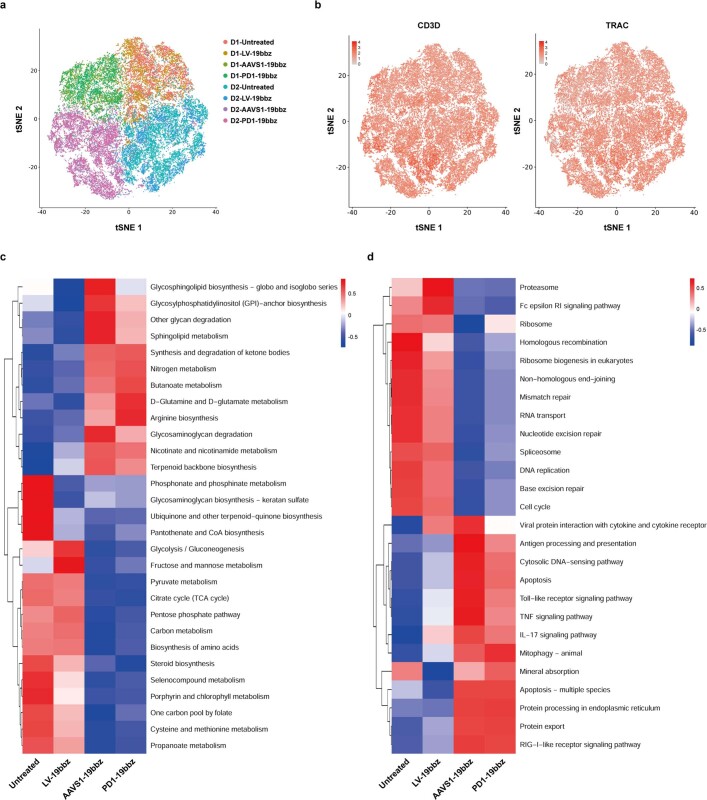
Single-cell RNA sequencing analysis of T cells collected shortly after preparation by different methods. Single-cell RNA sequencing was applied to analyse the characteristics of T cells collected 4 h after preparation by different methods. a, Overview of the 46,558 cells that passed QC for single-cell analysis. Cells are color coded by sample, respectively, in the tSNE plot. b, Expression of T cell marker genes (CD3D, TRAC) in the tSNE plots. c-d, Metabolism (c) and other (d) pathway activities in T cells were scored using the quantitative set analysis for gene expression (QuSAGE) method.

**Extended Data Fig. 11:**
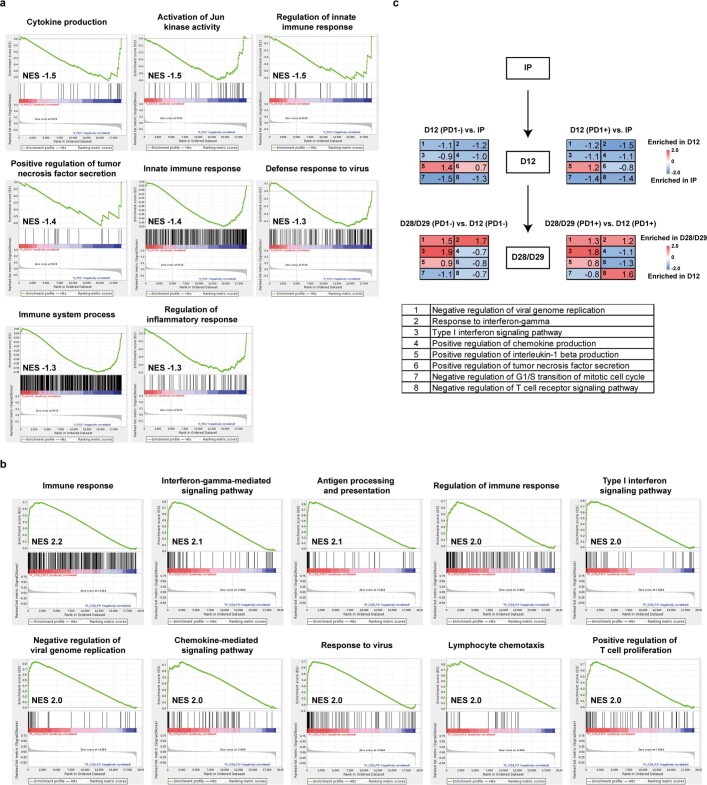
Gene set enrichment analysis of non-viral *PD1*-integrated CAR-T cells. a, Gene set enrichment analysis (GSEA) of CD8+ T cells comparing AAVS1-19bbz and PD1-19bbz cells. Enriched gene sets in PD1−19bbz cells and the normalized enrichment score (NES) are shown. b, GSEA of CD8+ T cells comparing the infusion products of patients with different prognosis (patient-1/patient-2, better prognosis; patient-3, worse prognosis). The top ten most enriched gene sets in patient-1/patient-2 group and the NES are shown. c, GSEA comparing different time point samples from patient-1 and patient-2 before and after infusion. CD8+ cells were analysed in the infusion products (IP). CD8+/CAR+/PD1− (left) and CD8+/CAR+/PD1+ (right) cells were analysed in D12 and D28/D29 samples after infusion, respectively. The serial numbers and names of enriched gene sets and the NES are shown. Positive values of the NES (red) represented the enrichment of gene sets in D12 (vs. IP) and D28/D29 (vs. D12) samples. Negative values of the NES (blue) represented the enrichment of gene sets in IP (vs. D12) and D12 (vs. D28/D29) samples. The patient-3 samples were not subjected to this analysis owing to an unreliable low CAR+ cell number in D28 sample.

**Extended Data Fig. 12:**
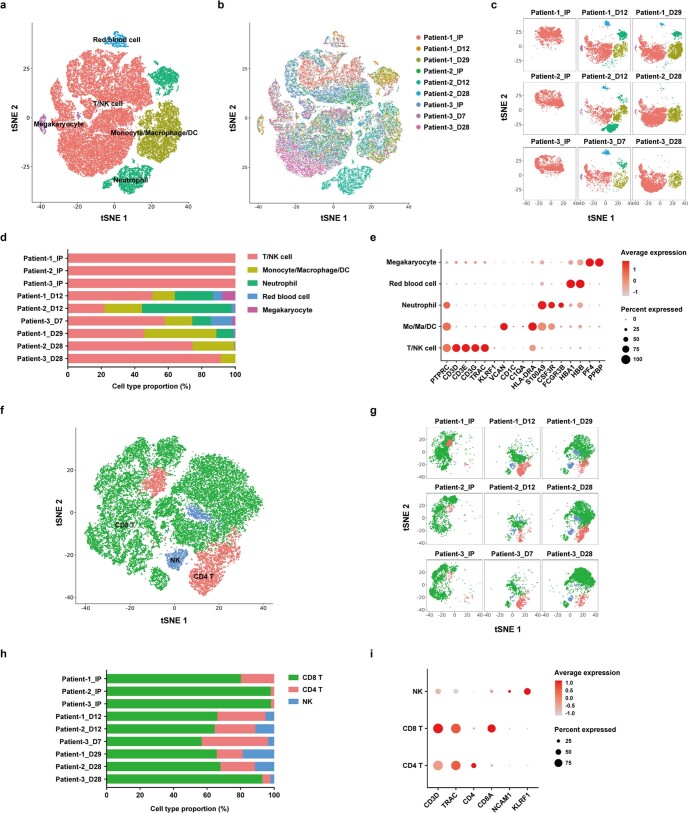
Landscape of cell types in single-cell RNA sequencing analysis of patient samples. a-b, Overview of the 54,774 cells that passed QC for single-cell analysis. Cells are color coded by cell type (a) and sample (b), respectively, in the tSNE plots. c, tSNE plots showing cell clusters in each sample. d, Proportion of cell types in each sample. e, Bubble heat map showing marker gene expression for different cell types. f, Overview of the 36,201 cells in the T/NK cell cluster. Cells are color coded by cell type in the tSNE plot. g, tSNE plots showing subtypes in the T/NK cell cluster in each sample. h, Proportion of subtypes in the T/NK cell cluster in each sample. i, Bubble heat map showing marker gene expression for different subtypes in the T/NK cell cluster. Source data

**Extended Data Fig. 13:**
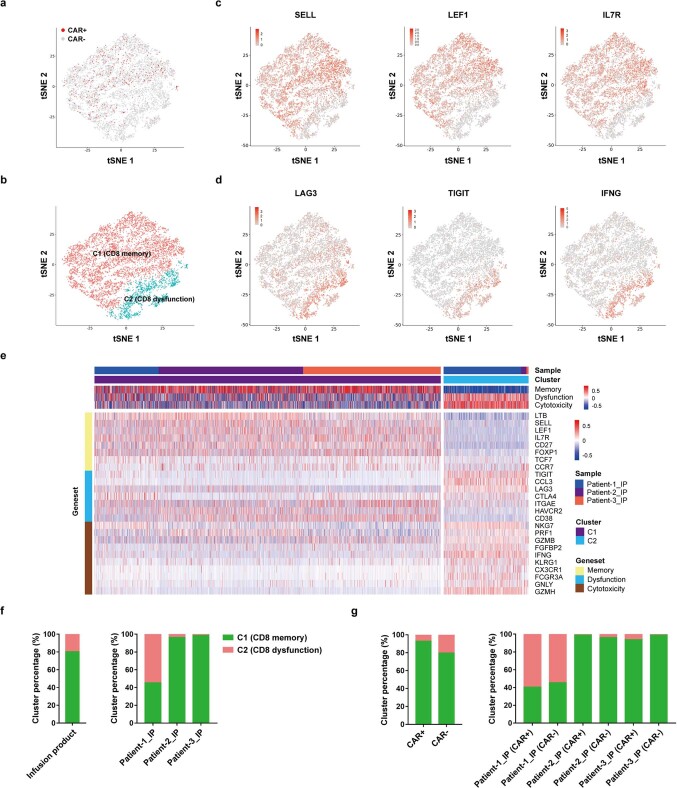
Proportion of CD8 memory and dysfunction clusters in infusion products. CD8+ T cells were analysed in the infusion products of three patients. a, Distribution of CAR+ and CAR− cells in the tSNE plot. b, tSNE plot showing two clusters in the infusion products. C1 and C2 were generated by clustering CD8 memory and dysfunction/cytotoxicity marker genes, respectively. c, Expression of representative CD8 memory genes (SELL, LEF1, IL7R) in the tSNE plots. d, Expression of representative CD8 dysfunction/cytotoxicity genes (LAG3, TIGIT, IFNG) in the tSNE plots. e, Heat map showing scaled expression of memory, dysfunction and cytotoxicity genes in two CD8+ T cell clusters. The GSVA scores of CD8 memory, dysfunction and cytotoxicity signatures are shown at the top. f, Percentages of C1 and C2 in mixed and individual samples of infusion products. g, Comparison of C1 and C2 proportion between CAR+ and CAR− cells in mixed and individual samples of infusion products. Source data

**Extended Data Fig. 14:**
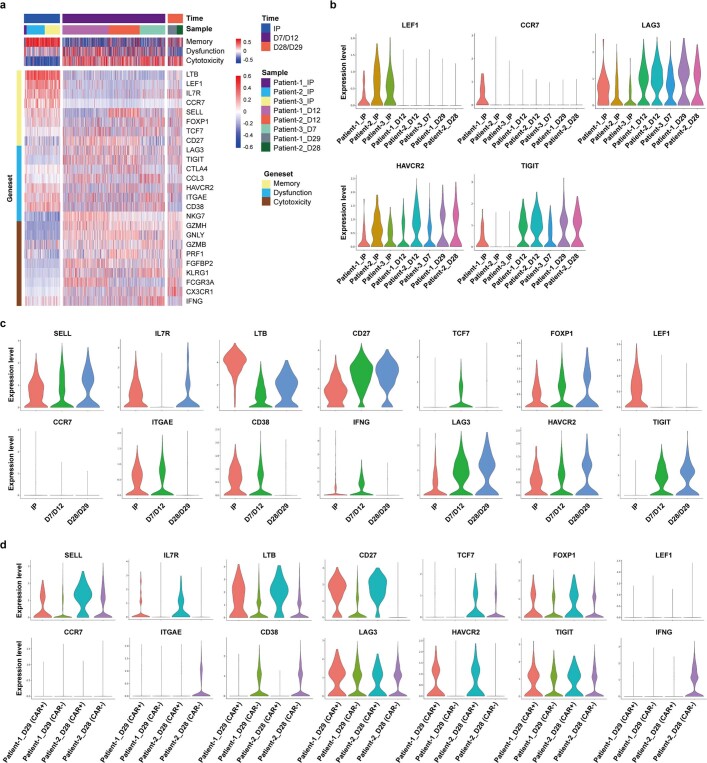
Expression of CD8 memory and dysfunction/cytotoxicity genes in non-viral *PD1*-targeted CAR-T cells before and after infusion. a, Heat map showing scaled expression of memory, dysfunction and cytotoxicity genes in CD8+/CAR+ cells from three patients before and after infusion. The GSVA scores of CD8 memory, dysfunction and cytotoxicity signatures are shown at the top. b,c, Violin plots showing the expression of memory and dysfunction/cytotoxicity genes in CD8+/CAR+ cells from individual (b) and mixed (c) samples of three patients before and after infusion. d, Violin plots showing the expression of memory and dysfunction/cytotoxicity genes in CD8+/CAR+ and CD8+/CAR− cells from two patients after 28 or 29 days infusion. The data of the patient-3 sample taken after 28 days treatment is excluded from mixed samples and not shown individually owing to an unreliable low CAR+ cell number.

**Extended Data Fig. 15:**
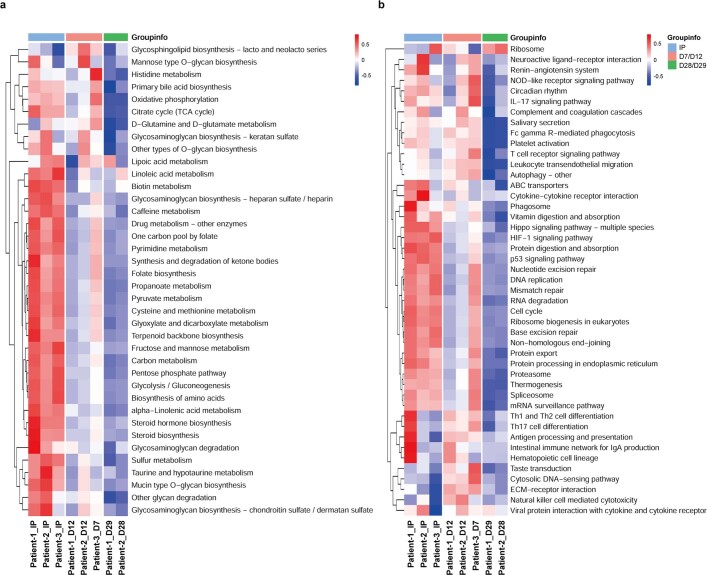
Pathway activities in non-viral *PD1*-integrated CAR-T cells before and after infusion. a-b, Metabolism (a) and other (b) pathway activities were scored using the QuSAGE method in CD8^+^/CAR^+^ cells from three patients before and after infusion. The data of patient-3 sample after 28 days treatment is not shown owing to an unreliable low CAR^+^ cell number.

To further understand the characteristics of non-viral, *PD1*-integrated CAR-T cells, single-cell RNA sequencing (scRNA-seq) was carried out in CAR-T cells prepared in parallel by different methods (Extended Data Fig. 8a–f). To unravel the features of each kind of CAR-T cell, two clusters were defined by using CD8^+^ memory and dysfunction/cytotoxicity marker genes and the expression of a wide range of memory, dysfunction and cytotoxicity genes^33–35^ was analysed (Extended Data Figs. 8h–j and 9a and Supplementary Table 9). Notably, the percentage of cells in the CD8^+^ memory cluster was significantly higher among cells with non-viral, gene-specific targeting (PD1-19bbz, AAVS1-19bbz), regardless of the integration site (Fig. 4a and Extended Data Figs. 8g and 9b–e). The differences caused by the distinct production methods were further uncovered by scRNA-seq of T cells collected shortly after their preparation (Extended Data Fig. 10). In comparison with AAVS1-19bbz cells, interference of PD1 conferred PD1-19bbz cells with an increased immune response capability (Fig. 4b and Extended Data Fig. 11a). Next, we conducted scRNA-seq to delineate the properties of PD1-19bbz cells before and after infusion using three patient samples (Extended Data Fig. 12). In line with the preclinical data, a high proportion of the CD8^+^ memory cluster was detected in the infusion products (Extended Data Fig. 13 and Supplementary Table 10). We found that multiple gene sets associated with immune response were enriched in the infusion products of patients with better prognosis, thus showing a potential correlation between pre-infusion T cells and the effectiveness of CAR-T cell therapy, which was in line with a recent study^35^ (Extended Data Fig. 11b and Supplementary Tables 11 and 12). Then, we set out to understand the kinetics of gene expression in CD8^+^CAR^+^ cells throughout treatment. Sustained expression of some memory genes and attenuated expression of several dysfunction and cytotoxicity genes were specifically found in CAR^+^ cells after infusion (Fig. 4c,d, Extended Data Figs. 14 and 15 and Supplementary Table 13). In accordance with the in vitro data, gene set enrichment analysis (GSEA) showed that infused CAR^+^ cells with attenuated PD1 expression had a higher proliferation and immune response capability in vivo (Fig. 4e, Extended Data Fig. 11c and Supplementary Tables 14 and 15). Altogether, these scRNA-seq data showed that PD1-19bbz cells have an increased number of memory T cells and enhanced anti-tumour immune functions, thus giving a mechanistic explanation for their high efficacy.

## Discussion

CRISPR–Cas9-mediated HDR is becoming a usual method to facilitate precise integration of target sequences^36–38^. Here we generated gene-specific integrated CAR-T cells without using virus and demonstrate the feasibility of formal large-scale production for clinical application. To our knowledge, we are the first to demonstrate the safety and efficacy of non-viral, gene-specific targeted CAR-T cells in a clinical trial. Superior safety was found for patients with r/r B-NHL by using non-viral, *PD1*-integrated anti-CD19 CAR-T cells, with only a low incidence of mild CRS and without occurrence of neurological toxicity. Our results are also consistent with two recently reported clinical trials^39,40^ and, accordingly, further demonstrate the safety of CRISPR–Cas9 application in T cell therapy. On the other hand, we observed high-rate and durable CR. In particular, responses were found in two patients with high PD-L1 expression, although CD19^–^ relapse occurred in one patient later, thereby providing support for the advantage of PD1 interference in PD1-19bbz cells. Surprisingly, despite an unexpectedly low initial dose or a simultaneously low percentage of CAR^+^ cells caused by the early and still premature manufacturing process, CR was achieved in all three of these patients, which indicates that non-viral, *PD1*-targeted CAR-T cells have more potency to kill tumour cells.

Our preclinical and clinical data demonstrate that PD1-19bbz cells have high efficacy against tumour cells, which can be explained by two features. First, the scRNA-seq data showed that there is a high percentage of memory T cells among PD1-19bbz cells. Intriguingly, this characteristic existed in both types of non-viral, gene-specific integrated cells, no matter where the CAR sequence was inserted, suggesting that the electroporation method using exogenous linear dsDNA can eventually produce more memory T cells in infusion products. This superiority was supported by preclinical experiments showing that AAVS1-19bbz cells more potently eradicated Raji cells at a low infusion dose than LV-19bbz cells, and PD1-19bbz cells showed a stronger ability to eradicate tumour cells with high PD-L1 expression than LV-19bbz_PD1-KO cells, although *PD1* knockout was achieved in both cell types. Second, the GSEA results indicate that PD1 disruption confers PD1-19bbz cells with enhanced anti-tumour immune functions. This advantage was validated by the finding that PD1-19bbz cells more vigorously eliminated tumour cells in comparison with AAVS1-19bbz cells in mouse models. Notably, this superiority was observed even in tumour cells that had low PD-L1 expression. One possible explanation for this is that loss of PD1 could relieve the immune suppression caused by PD-L1 engagement on T cells, as reported recently^41^. Another is that other anti-tumour immune pathways, independent of PD-L1, were ameliorated by PD1 downregulation^42–44^. Moreover, given that inhibitory receptors that parallel PD1 function, such as LAG3, TIM3 and TIGIT, were still highly expressed in PD1-19bbz cells after clinical infusion, simultaneously intervening in multiple pathways holds promise to further augment the function of CAR-T cells.

In this study, we describe a new strategy to develop non-viral, gene-specific targeted CAR-T cells by CRISPR–Cas9. This technology is advanced owing to combining the advantages of both non-viral manufacturing processes and precise genome editing. As a two-in-one approach without using virus, the manufacturing procedure is simplified, with shortened preparation time, reduced production expenses and increased safety and efficacy of the CAR-T cell products. These advantages are important, especially for the generation of gene-modified CAR-T cells where virus preparation and genome editing processes are normally both required. Furthermore, locus-specific integration augments the homogeneity of the CAR-T cells and makes it possible to exploit versatile cell products. Importantly, we show the feasible application of this technology from bench to bedside and demonstrate its high safety and efficacy in a clinical trial. Thus, we propose an innovative CAR-T technology to break through the current barriers and show the considerable potential of CRISPR–Cas9-mediated non-viral, gene-specific targeted technology in cell therapy.

## Methods

### Clinical trial information and design

This study was a phase 1, open-label, single-arm clinical trial designed to evaluate the safety and efficacy of non-viral, *PD1*-integrated anti-CD19 CAR-T cells in treating aggressive r/r B-NHL. The clinical protocol has been registered at ClinicalTrials.gov (NCT04213469). The inclusion criteria were as follows: (1) age of 18 to 70 years; (2) diagnosis with CD19^+^ r/r B-NHL (stages III–IV); (3) life expectancy of >3 months; (4) Eastern Cooperative Oncology Group (ECOG) score of ≤2 and satisfactory major organ functions; and (5) a negative pregnancy test for women of reproductive potential and agreement to use birth control during the study. The exclusion criteria included (1) pregnancy or breast feeding; (2) refusal to use birth control during the next 2 years; (3) allo-haematopoietic stem cell transplantation within 6 months or previous treatment of graft-versus-host disease; (4) active autoimmune disease requiring immunosuppressive agents; (5) active infection; (6) history of other malignancies; and (7) ineligibility or lack of ability to comply with the study. To preliminarily assess the safety and effectiveness of this new CAR-T cell therapy, eight patients who had not previously been treated with CAR-T cell therapy were enrolled in the cohort with an infusion dose of 2 × 10^6^ CAR-T cells per kg. Because of the premature manufacturing process and individual variance, the cell number for three infusion products could not meet the planned dose requirement; thus, the actual infusion doses in these patients were lower than 1 × 10^6^ cells per kg (Extended Data Table 1 and Supplementary Table 5). This therapy included 3 d of lymphodepletion chemotherapy using combined fludarabine (25 mg m^–2^ from days −4 to −2) and cyclophosphamide (250 mg m^–2^ from days −3 to −2). CAR-T cell infusion was performed 2 d after the end of lymphodepletion chemotherapy and was followed by standard monitoring. The trial was approved by the institutional review board, and all patients provided written informed consent in accordance with the Declaration of Helsinki before enrolment. The clinical protocol was reviewed and approved by the Clinical Research Ethics Committee of the First Affiliated Hospital, College of Medicine, Zhejiang University (2020IIT(85)). Characteristics, clinical responses and prior therapies of the patients are shown in Extended Data Table 1 and Supplementary Table 6.

### Response assessment

Treatment response was assessed according to revised criteria of the Lugano classification. PET-CT scans and bone marrow biopsy were the major methods applied to evaluate lymphoma lesions. The response assessment criteria were as follows: (1) CR (complete remission): absence of clinical symptoms and PET-CT and bone marrow evidence associated with lymphoma; (2) PR (partial remission): lymphoma volume decrease of at least 50% without new lymphoma lesions or sustained bone marrow involvement; (3) PD (progressive disease): lymphoma volume increase of at least 50% or onset of new lymphoma lesions; (4) SD (stable disease): a condition that did not meet the criteria for CR, PR or PD. Response duration was calculated from the first documentation of response until disease progression, initiation of off-study treatment or the last documentation of ongoing response.

### Assessment and grading of CRS

Serum cytokines including IL-2, IL-4, IL-6, IL-10, IFNγ, TNFα and IL-17A were assessed with the Human Th1/Th2/Th17 CBA kit (BD Biosciences) within 1 month of infusion. CRS was assessed and graded according to the National Cancer Institute Common Terminology Criteria for Adverse Events (NCI-CTCAE) version 5.0 in combination with other methods^45^. Among the eight patients, only patient 6 was treated with an IL-6 antagonist, tocilizumab.

### Assessment and grading of neurological toxicity

Neurological toxicities were assessed and graded according to NCI-CTCAE version 5.0. Once CRS symptoms such as pyrexia, hypotension and capillary leak or other types of AEs were observed, the patient would be closely monitored for signs of neurological toxicity, such as seizure, tremor, encephalopathy or dysphasia.

### Assessment and grading of AEs

Patients were inpatients and were closely monitored after receiving lymphodepletion chemotherapy and CAR-T cell infusion. Physical and clinical laboratory examinations were documented during hospitalization to evaluate the toxicity of the treatment. AEs were graded using NCI-CTCAE version 5.0. All AEs are summarized in Supplementary Table 7. During hospitalization, any AEs that occurred after CAR-T cell infusion were recorded. Severe AEs, except a decrease in lymphocyte counts caused by lymphodepletion chemotherapy, were required to be reported to the Medical Ethics Committee of the First Affiliated Hospital, College of Medicine, Zhejiang University within 24 h of occurrence. One month after infusion, patients underwent follow-up and were monitored for disease progression and toxicity once a month thereafter.

### Immunohistochemistry

Immunohistochemistry (IHC) analysis was undertaken on formalin-fixed, paraffin-embedded tissue sections. In brief, after sections were deparaffinized in xylene and rehydrated in a graded alcohol series, endogenous peroxidase was blocked with 3% hydrogen peroxide. Antigen retrieval was performed using EDTA buffer (pH 9.0). After rinsing sections in PBS, antibodies against human CD19 (Biolynx) and PD-L1 (Agilent) were used for IHC staining. Staining was carried out on an automated immunostainer (Leica Bond-III, Dako Autostainer Link 48) using a Bond Polymer Refine Detection system.

### Cell lines

293T and Nalm-6 cells were purchased from the American Type Culture Collection, and Raji cells were purchased from the Cell Bank of the Chinese Academy of Sciences. All cell lines were authenticated by short-tandem-repeat profiling. 293T cells were maintained in DMEM (Gibco) supplemented with 10% FBS (Thermo Fisher). Nalm-6 and Raji cells were maintained in RPMI-1640 (Thermo Fisher) supplemented with 10% FBS (Thermo Fisher). A Raji cell line stably expressing firefly luciferase (ffLuc) was established by lentiviral infection. Raji cells stably expressing PD-L1 were generated using a lentiviral vector containing a co-expression cassette for PD-L1 and ffLuc. All stable cell lines underwent selection with puromycin. All cell lines were regularly tested to ensure they were free of mycoplasma contamination.

### Isolation and expansion of human primary T cells

Fresh peripheral blood mononuclear cells (PBMCs) from healthy donors were provided by the First Affiliated Hospital, College of Medicine, Zhejiang University and Shanghai SAILY Biological Technology Co., Ltd. Recruitments of healthy human blood donors was approved by the Clinical Research Ethics Committee of the First Affiliated Hospital, College of Medicine, Zhejiang University and by Shanghai Zhaxin Traditional Chinese Ethics Committee and Western Medicine Hospital. All donors signed an informed consent form. Fresh PBMCs from patients were collected by apheresis. PBMCs were isolated by density gradient centrifugation using Ficoll (Sigma-Aldrich). T cells were enriched through magnetic separation using anti-CD4 and anti-CD8 microbeads (Miltenyi Biotec) and activated with T Cell TransAct (Miltenyi Biotec). T cells were cultured in X-VIVO 15 medium (Lonza) supplemented with 2% human AB serum or CTS Immune Cell Serum Replacement (Thermo Fisher) and recombinant human IL-2 (100 U ml^–1^), IL-7 (5 ng ml^–1^) and IL-15 (5 ng ml^–1^). Cells were collected once cell number reached the requirement for administration and then washed, formulated and cryopreserved.

### Construction of CAR cassette

The anti-CD19 CAR cassette was composed of the single-chain variable fragment derived from clone FMC63, the extracellular domain and transmembrane regions of CD8α, the intracellular domain of 4-1BB (CD137) and the intracellular domain of CD3ζ. Transcription of the CAR element was driven by an EF1α promoter and terminated by an SV40 poly(A) signal sequence. In this study, the same anti-CD19 CAR cassette was used in different constructs.

### Ribonucleoprotein and linear dsDNA production

A two-component sgRNA was chemically synthesized (GenScript) and resuspended with TE buffer. Ribonucleoproteins (RNPs) were produced by complexing one sgRNA and recombinant spCas9 (Thermo Fisher) for 10 min at room temperature. RNPs were subjected to electroporation immediately after complex formation. For linear dsDNA production in preclinical experiments, plasmids containing an mTurquoise2 or anti-CD19 CAR sequence flanked by homology arms were first constructed. Linear dsDNA was then obtained by restriction endonuclease digestion and purified by TIANgel DNA Purification kit (Tiangen Biotech). The sgRNAs used were as follows: AAVS1 sgRNA (5′-AGAGCUAGCACAGACUAGAG-3′; chr19:55115996, intron 1 of *PPP1R12C*), PD1 sgRNA (site 1) used for the preparation of PD1-19bbz and LV-19bbz_PD1-KO cells (5′- CGACUGGCCAGGGCGCCUGU-3′; chr2:241858824, exon 1 of *PD1*), PD1 sgRNA (site 2) (5′-GGGCGGUGCUACAACUGGGC-3′; chr2:241858788, exon 1 of *PD1*), TRAC sgRNA (5′-AGAGCAACAGTGCTGTGGCC-3′; chr14:22547693, exon 1 of *TRAC*), B2M sgRNA (5′-GAGTAGCGCGAGCACAGCTA-3′; chr15:44711569, exon 1 of *B2M*).

### Human primary T cell electroporation

Electroporation was performed 2–3 d after T cell stimulation. For preparation of non-viral, gene-specific targeted CAR-T cells from healthy donors and some patients, the procedure was conducted following the manufacturer’s instructions using a Lonza 4D electroporation system. In brief, 2.5 × 10^6^ to 1.5 × 10^7^ prewashed T cells were resuspended in 100 μl electroporation buffer P3. Meanwhile, RNPs were prepared, followed by mixture with the DNA template (1.5–20 μg). Cells in electroporation buffer were then added and moved into electroporation cuvettes. Programme EO115 was chosen for electroporation. After electroporation, cells were immediately supplemented with prewarmed medium and transferred out of the electroporation cuvettes.

To prepare PD1-19bbz cells for some patients, we followed the manufacturer’s instructions using the GT Flow Transfection System (MaxCyte). In brief, prewashed T cells were resuspended in MaxCyte electroporation buffer. Meanwhile, RNPs were prepared, followed by mixture with the DNA template. Cells in electroporation buffer were then added and moved into a static processing assembly (CL-1.1). After electroporation, cells were transferred for recovery and then added to culture medium. The reaction conditions were scaled up according to those used in the Lonza 4D electroporation system.

### CAR-T cell generation by lentivirus

The CAR sequence was cloned into the pCDH lentiviral vector backbone containing an EF1α promoter. Lentivirus was produced by transfecting 293T cells with CAR plasmid, pMD2.G and psPAX2 using polyethylenimine. Virus-containing supernatants were collected after 3 d to infect primary human T cells stimulated for 2–3 d. LV-19bbz_PD1-KO cells were prepared by lentiviral infection followed by electroporation using recombinant spCas9 (Thermo Fisher) and PD1 sgRNA after 2 d.

### Indel percentage analysis

Genomic DNA was obtained using a Genomic DNA Purification kit (Thermo Fisher). Fragments containing indel sites were amplified by PCR using specific primers and purified by TIANgel DNA Purification kit (Tiangen Biotech). DNA sequencing was carried out, and the indel percentage was measured by ICE analysis (Synthego). The primers used were as follows: AAVS1-Forward, 5′-CACCACGTGATGTCCTCTGA-3′; AAVS1-Reverse, 5′-CCGGCCCTGGGAATATAAGG-3′; PD1-Forward, 5′-CCACGTGGATGTGGAGGAAG-3′; PD1-Reverse, 5′-CCACACAGCTCAGGGTAAGG-3′.

### Genotyping of PD1-19bbz cells

PD1-19bbz cells were prepared using T cells from two independent healthy donors. Some CAR^+^ cells were sorted by fluorescence-activated cell sorting (FACS). Genomic DNA was isolated using the Genomic DNA Purification kit (Thermo Fisher). Semiquantitative PCR using three primers in one reaction was carried out. One forward primer (5′-CCCTGCAACTGATGGTGACT-3′) specifically binds to the anti-CD19 CAR cassette. A reverse primer (5′-TCACAGTGTACACAGAGGGC-3′) recognizes a genomic sequence outside the right homology arm. Another forward primer (5′-GACAGTTTCCCTTCCGCTCA-3′) recognizes a genomic sequence within the left homology arm. The intensity ratio of wild-type/indel and CAR-specific bands in unsorted and sorted cells was calculated by densitometry quantification to genotype PD1-19bbz cells, where *A* was the wild-type/indel percentage in sorted cells, *B* was the CAR percentage in sorted cells, *C* was the wild-type/indel percentage in unsorted cells and *D* was the CAR percentage in unsorted cells. The genotype percentages in samples were calculated with the following equations: wild-type/indel (%) = (*B* × *C* – *A* × *D*)/*B* × 100%, heterozygous (%) = 2 × *A* × *D*/*B* × 100% and homozygous (%) = (*B* – *A*) × *D*/*B* × 100%.

### Deep sequencing

Deep sequencing (10,000× coverage) was carried out to detect indels at the *PD1* on-target site and 29 top-ranked off-target sites predicted by the Benchling CRISPR tool or to validate possible indels preliminarily indicated by whole-genome sequencing or off-target events at 24 top-ranked potential sites identified by iGUIDE in one representative infusion product (patient 2). Deep sequencing (50,000× coverage) was also performed to validate off-target events at the *PHACTR1* site in different infusion products. Genomic DNA from untreated T cells and infusion products was isolated using the Genomic DNA Purification kit (Thermo Fisher). Fragments containing indel sites were amplified by PCR using specific primers and subjected to sequencing on a Hi-TOM platform as described previously^46^. Deep sequencing was carried out by the XI’AN CyanSnow Gene Company.

### Whole-genome sequencing

Genomic DNA from untreated T cells and the infusion product of patient 2 was extracted using the Blood & Cell Culture DNA kit (Qiagen) according to the manufacturer’s instructions and subjected to library construction. Sequencing libraries were generated using the TruSeq Nano DNA HT sample preparation kit (Illumina) following the manufacturer’s recommendations, and index codes were added to attribute sequences to each sample. These libraries including untreated and edited T cells were sequenced on the HiSeq platform (Illumina) with 100× coverage. BWA (Burrows–Wheeler aligner)^47^ was used to align clean reads for each sample against the reference genome (settings: mem -t 5 -M -R). Alignment files were converted to BAM files using SAMtools^48^ (settings: -bS -t). In addition, potential PCR duplications were removed using the sambamba command ‘markdup’. If multiple read pairs had identical external coordinates, only the pair with the highest mapping quality was retained. Indels (<50 bp) were calculated and identified with MPILEUP in SAMtools^48^ and were processed using picard-tools. To reduce the indel detection error rate, we filtered out indels for which the number of supporting reads was less than 4, the quality value (MQ) was less than 30 and QUAL was less than 20. Indels were filtered, with those near other variants and within the pseudoautosomal region (PAR) removed. Whole-genome sequencing was carried out by Novogene Co., Ltd.

We used Cas-OFFinder (http://www.rgenome.net/cas-offinder/) to predict potential off-target sites. Any sequence, followed by an NRG protospacer adjacent motif, having no more than five mismatches (a bulge penalty equals two base mismatches) with the PD1 sgRNA was screened with a total of 2,219 sites (not including those around the on-target site) identified. Indels exclusively detected in the edited sample and located around potential off-target sites were searched. No indel events were found within 15 bp upstream and downstream (±15 bp) of the sites. Indel events were detected within 200 bp upstream and downstream (±200 bp) of eight sites. Deep sequencing with 10,000× coverage was performed to validate these indel events.

### iGUIDE

The locations of off-target cleavage sites were mapped using iGUIDE^31^, an improvement of the GUIDE-seq method^32^. In brief, stimulated T cells were mixed with RNP (spCas9–PD1 sgRNA) and a protected double-stranded oligodeoxynucleotide (dsODN) and were then subjected to electroporation. Genomic DNA was isolated as described previously after 6 d of cell culture. Next, DNA was cleaved by sonication, adaptors were ligated to free DNA ends and PCR was performed using primers that annealed to the adaptor and dsODN. PCR products were analysed by Illumina sequencing, and reads were mapped to the human genome. Given the presence of a flanking reporter dsODN sequence in correct priming, reads resulting from mispriming could be identified and filtered out. The sequencing data were analysed using the iGUIDE pipeline, available at https://github.com/cnobles/iGUIDE. The gene lists used are available at http://bushmanlab.org/assets/doc/allOnco_Feb2017.tsv and http://bushmanlab.org/assets/doc/humanLymph.tsv.

### scRNA-seq

Various CAR-T cells (LV-19bbz, LV-19bbz_PD1-KO, AAVS1-19bbz, PD1-19bbz) were prepared in parallel using T cells from two patients (patients 1 and 2) by different methods and then collected after 9 d. Several CAR-T cell types (LV-19bbz, AAVS1-19bbz, PD1-19bbz) were prepared in parallel using T cells from two healthy donors (D1 and D2) by different methods and immediately collected after 4 h. Fresh PBMCs from patients were collected by apheresis at the peak (day 7 or 12) and stable (day 28 or 29) stages of CAR-T cell expansion after infusion and then isolated by density gradient centrifugation using Ficoll (Sigma-Aldrich). Differently prepared CAR-T cells, infusion products and PBMCs from three patients (patients 1–3) were subjected to scRNA-seq.

scRNA-seq libraries were generated using a 10x Genomics Chromium Controller instrument and Chromium Single-Cell 3′ V3.1 reagent kits (10x Genomics). In brief, cells were concentrated to 1,000 cells per μl and approximately 7,000 cells were loaded into each channel to generate single-cell Gel Bead-In-Emulsions (GEMs), resulting in mRNA barcoding of an expected 5,000 single cells for each sample. After the reverse transcription step, GEMs were broken and barcoded cDNA was purified and amplified. The amplified barcoded cDNA was fragmented, A-tailed, ligated with adaptors and amplified by index PCR. The final libraries were quantified using the Qubit High-Sensitivity DNA assay (Thermo Fisher), and the size distribution of the libraries was determined using a High-Sensitivity DNA chip on a Bioanalyzer 2200 (Agilent). All libraries were sequenced on an Illumina sequencer using a 150-bp paired-end run.

We applied fastp^49^ with default parameter to filter out the adaptor sequence and remove low-quality reads to achieve clean data. Feature-barcode matrices were then obtained by aligning reads to the human genome (GRCh38 version 91, Ensembl) using CellRanger v3.1.0. We applied downsampled analysis among the samples sequenced according to the mapped barcoded reads for each cell of each sample to achieve the aggregated matrix. Cells with over 200 expressed genes and a mitochondrial unique molecular identifier (UMI) rate below 20% passed the cell quality filtering, and mitochondrial genes were removed from the expression table.

The Seurat package (v.3.1.4; https://satijalab.org/seurat/) was used for cell normalization and regression based on the expression table according to the UMI counts of each sample and the mitochondrial rate to obtain scaled data. Principal-component analysis (PCA) was performed on the basis of the scaled data with the top 2,000 most highly variable genes and the top ten principal components used for *t*-SNE construction and UMAP construction. Using the graph-based cluster method, we acquired the unsupervised cell cluster result on the basis of the top ten principal components from PCA and calculated the marker genes by the FindAllMarkers function with the Wilcoxon rank-sum test algorithm under the following criteria: (1) ln(fold change) > 0.25; (2) *P* < 0.05; (3) min.pct > 0.1. To characterize the relative activation of a given gene set such as the KEGG pathway ‘memory, dysfunction and cytotoxicity’ as described previously, we used QuSAGE (v.2.16.1)^50^ to calculate the score for each cluster/sample and GSVA (v.1.32.0)^51^ to calculate the score for each cell. GSEA (http://broadinstitute.org/gsea) was used to analyse differentially enriched gene sets between samples. scRNA-seq and data analysis were performed by NovelBio Bio-Pharm Technology Co., Ltd.

### Flow cytometry

CAR and membrane protein expression was determined by flow cytometry. Cells were prewashed and incubated with antibodies for 30 min on ice. After washing twice, samples were run on an LSRFortessa (BD Biosciences) or DxFLEX Flow Cytometer (Beckman Coulter) and analysed with FlowJo software. The following antibodies were used: FITC anti-human CD3, APC anti-human CD69, APC anti-human CD137, APC anti-human CD25, APC anti-human PD1, APC anti-human LAG3, APC anti-human TIM3, BV421 anti-human CD45RO, APC anti-human CD62L, APC anti-human CD3, FITC anti-human CD19, FITC anti-human CD4, APC anti-human CD4, APC anti-human CD8, BV421 anti-human CD45 (all from BioLegend), PerCP-Cy5.5 anti-human CD4, BV421 anti-human CD8, PerCP-Cy5.5 anti-human CD45 (all from BD Biosciences) and APC anti-human PD-L1 (Thermo Fisher). For detection of CAR expression, biotinylated human CD19 (amino acids 20–291; ACRO Biosystems) and PE streptavidin (BioLegend) were added sequentially or PE-labelled human CD19 (amino acids 20–291; ACRO Biosystems) was used. For some experiments, CAR-T cells were co-cultured with target cells at an effector/target ratio of 1:1 (Raji cells) or 1:2 (PD-L1-expressing Raji cells) for 24 h before collection. For detection in clinical samples, peripheral blood cells were stained with antibodies, followed by addition of Lysis Buffer (BD Biosciences) before being run. The percentage of CAR^+^ cells was analysed in CD45^+^CD3^+^ gated cells.

### CAR copy number analysis by qPCR

Blood samples were collected before and after CAR-T cell infusion. Lysis Buffer (BD Biosciences) was added, and genomic DNA was acquired using the Genomic DNA Purification kit (Thermo Fisher). A seven-point standard curve was generated by using 5 × 10^0^ to 5 × 10^6^ copies per μl of lentiviral vector DNA containing the 19bbz sequence. TaqMan qPCR assays were performed to measure CAR copy number in peripheral blood cells. qPCR was run on a QuantStudio 3 Real-Time PCR System (Thermo Fisher). Each sample was analysed in triplicate. Primers specifically targeting the 19bbz sequence were as follows: forward, 5′-GCTGTAGCTGCCGATTTCCA-3′; reverse, 5′-GGTTCTGGCCCTGCTTGTAC-3′; probe, 5′-AGTGAAGTTCAGCAGGAGCGCAGACG-3′.

### Antigen stimulation and proliferation of CAR-T cells

As antigen for stimulation, Raji or PD-L1-expressing Raji cells were pretreated with mitomycin C (50 μg ml^–1^) for 90 min at 37 °C. CAR-T cells were co-cultured with target cells at an effector/target ratio of 1:1 (Raji cells) or 1:2 (PD-L1-expressing Raji cells) for 3–4 d per stimulation. The number of CAR^+^ cells was determined by multiplying the total viable cell number and the percentage of CAR^+^ cells. Cell viability was measured by Trypan blue staining.

### CellTrace Violet proliferation assays

AAVS1-19bbz cells were labelled with CellTrace Violet (Thermo Fisher) according to the manufacturer’s instructions. Raji cells were pretreated with mitomycin C (50 μg ml^–1^) for 90 min at 37 °C. CAR-T cells and target cells were mixed at an effector/target ratio of 1:1. After 5 d, cells were collected and run on an LSRFortessa (BD Biosciences).

### Bead-based immunoassays

In preclinical experiments, CAR-T cells were co-cultured with Raji or PD-L1-expressing Raji cells at an effector/target ratio of 1:1 (Raji cells) or 1:2 (PD-L1-expressing Raji cells) in medium without exogenous cytokines. The supernatant was collected after 24 h, and cytokines were measured using LEGENDplex bead-based immunoassays (BioLegend) according to the manufacturer’s instructions.

### ELISAs

For in vitro evaluation of infusion products, CAR-T cells were co-cultured with Nalm-6 cells at an effector/target ratio of 1:1 in medium without exogenous cytokines. The supernatant was collected after 18–24 h, and IFNγ secretion was measured using the Human IFN-γ ELISA kit (StemCell) according to the manufacturer’s instructions.

### Flow cytometry-based cytotoxicity assays

AAVS1-19bbz cells were co-cultured with Raji cells at an effector/target ratio of 1:1 for 18 h. Flow cytometry was used to detect residual tumour cells by staining with APC anti-human CD3 and FITC anti-human CD19 antibodies. Cells were enumerated using CountBright Absolute Counting Beads (Thermo Fisher) following the manufacturer’s instructions.

### LDH cytotoxicity assays

CAR-T cells were co-cultured with Nalm-6, Raji or PD-L1-expressing Raji cells at the indicated effector/target ratios. Cytotoxicity was measured by release of LDH using the CytoTox 96 Non-Radioactive Cytotoxicity Assay (Promega) according to the manufacturer’s instructions.

### In vivo mouse experiments

All animal experiments were conducted in compliance with the Guide for the Care and Use of Laboratory Animals (2011) issued by the National Research Council (USA), Laboratory Animal Administration Regulation (2017) issued by the National Science and Technology Committee (China) and the laboratory animal administration regulations (Shanghai, Jiangsu). The care and use of animals were reviewed and approved by the Institutional Animal Care and Use Committee (IACUC) of the East China Normal University Center for Animal Research or InnoStar Bio-tech Nantong Co., Ltd. For the experiment comparing the LV-19bbz and AAVS1-19bbz groups at a high infusion dose, 6- to 8-week-old B-NDG (NOD.CB17-*Prkdc*^*scid*^*Il2rg*^*tm1*^/Bcgen) male mice (Biocytogen) were injected intravenously with 2 × 10^5^ ffLuc-transduced Raji cells. Subsequently, 2 × 10^6^ CAR-T cells were administered intravenously after 5 d. For the experiment comparing the LV-19bbz, AAVS1-19bbz and PD1-19bbz groups at a low infusion dose, 6- to 9-week-old NCG (NOD/ShiLtJGpt-*Prkdc*^*em26Cd52*^*Il2rg*^*em26Cd22*^/Gpt) female mice (GemPharmatech) were injected intravenously with 2 × 10^5^ ffLuc-transduced Raji cells. Subsequently, 1 × 10^6^ CAR-T cells were administered intravenously after 5 d. For other experiments, 6- to 9-week-old NCG female mice (GemPharmatech) were inoculated intravenously with 5 × 10^5^ ffLuc-transduced PD-L1-expressing Raji cells. Subsequently, 1 × 10^6^ or 2 × 10^6^ CAR-T cells were injected intravenously after 5 d. Bioluminescence images were acquired and analysed using the IVIS Imaging System and software (PerkinElmer). Mice were randomized on the basis of tumour radiance before CAR-T cell injection. Mice were killed according to the experimental protocols or when they met prespecified endpoints defined by the IACUC. Animal technicians were blinded to expected outcomes. The experiments were performed in the East China Normal University Center for Animal Research or InnoStar Bio-tech Nantong Co., Ltd.

### Statistics

Experimental data are presented as the mean ± s.d. or the mean ± s.e.m. as described in the figure legends. Data were analysed by one-way ANOVA, two-way ANOVA or log-rank Mantel–Cox test as indicated using GraphPad software. *P* < 0.05 was considered statistically significant. Asterisks are used to indicate significance: ****P < 0.0001, ***P < 0.001, **P < 0.01, *P < 0.05. NS, not significant.

### Reporting summary

Further information on research design is available in the Nature Research Reporting Summary linked to this article.

## Online content

Any methods, additional references, Nature Research reporting summaries, source data, extended data, supplementary information, acknowledgements, peer review information; details of author contributions and competing interests; and statements of data and code availability are available at 10.1038/s41586-022-05140-y.

### Supplementary information

Supplementary Information This file contains Supplementary Tables 1–7.

Reporting Summary

Supplementary Table 8 Off-target sites identified by iGUIDE.

Supplementary Table 9 Differentially expressed genes in CD8^+^ T cells between different samples.

Supplementary Table 10 Differentially expressed genes between two CD8^+^ T cell clusters in three infusion products.

Supplementary Table 11 Differentially expressed genes in CD8^+^ T cells between patient 1/patient 2 and patient 3 infusion products.

Supplementary Table 12 GSEA of CD8^+^ T cells between the infusion products of patients with different responses (enriched gene sets in the patient 1/patient 2 group).

Supplementary Table 13 Differentially expressed genes in CD8^+^CAR^+^ T cells between different samples before and after infusion.

Supplementary Table 14 Differentially expressed genes between CD8^+^CAR^+^PD1^+^ and CD8^+^CAR^+^PD1^–^ cells in day 7/12 samples after infusion.

Supplementary Table 15 GSEA between CD8^+^CAR^+^PD1^+^ and CD8^+^CAR^+^PD1^–^ cells in day 7/12 samples after infusion (enriched gene sets in CD8^+^CAR^+^PD1^–^ cells).

### Source data

Source Data Fig. 1

Source Data Fig. 2

Source Data Fig. 3

Source Data Fig. 4

Source Data Extended Data Fig. 1

Source Data Extended Data Fig. 2

Source Data Extended Data Fig. 3

Source Data Extended Data Fig. 4

Source Data Extended Data Fig. 5

Source Data Extended Data Fig. 7

Source Data Extended Data Fig. 8

Source Data Extended Data Fig. 9

Source Data Extended Data Fig. 12

Source Data Extended Data Fig. 13

## Figures and Tables

**Extended Data Table 1:**
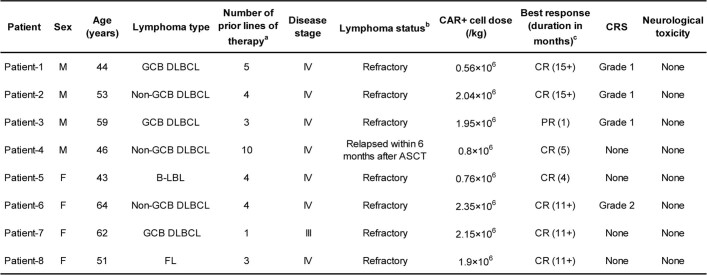
Patient characteristics, clinical responses and adverse events

ASCT, autologous stem cell transplantation; B-LBL, B-cell lymphoblastic lymphoma; CR, complete remission; DLBCL, diffuse large B cell lymphoma; F, female; FL, follicular lymphoma; GCB, germinal center B cell; M, male; PR, partial remission. ^a^All prior lines of therapy for each patient are listed in Supplementary Table 6. None of the patients were treated with CAR-T cell therapy previously. ^b^Disease was defined as refractory if a patient did not achieve partial or complete remission after the most recent chemotherapy. ^c^Best response was defined as the best response that a patient achieved after CAR-T cell infusion. Response duration is the time from the first documentation of response, until disease progression, initiation of off-study treatment or the last documentation of ongoing response. The + symbol indicates an ongoing response.

## Data Availability

scRNA-seq data have been deposited in the GEO database (GSE166352, GSE186596, GSE201035). Whole-genome sequencing, iGUIDE and deep sequencing data have been deposited in the SRA database (PRJNA774073, PRJNA772163, PRJNA772700, PRJNA772894, PRJNA772887, PRJNA772893). Source data are provided with this paper.
