# Supplementary material for: Non-viral, specifically targeted CAR-T cells achieve high safety and efficacy in B-NHL
Source: Nature. 2022 Aug 31;609(7926):369–74. doi: 10.1038/s41586-022-05140-y (PMC9452296; doi:10.1038/s41586-022-05140-y)
Supplement: Supplementary file 1 — This file contains Supplementary Tables 1–7. [file 41586_2022_5140_MOESM1_ESM.pdf]

nature portfolio

https://doi.org/10.1038/s41586-022-05140-y

Supplementary information

Non-viral, specifically targeted CAR-T cells achieve high safety and efficacy in B-NHL

In the format provided by the authors and unedited

Supplementary Table 1

Deep sequencing analysis of mutations at the *PD1* on-target site

Target	Gene ID	Edit Site	Sequence	Mutation Type	Ratio (%)
On	PDCD1	chr2:+:241858824 (exon 1)	CGACTGGCCAGGGCGCC-GTGGG	1 bp deletion (frameshift)	17.03%
			CGACTGGCCAGGGCGC-TGTGGG	1 bp deletion (frameshift)	14.61%
			CGACTGGCCAGGGCG--TGTGGG	2 bp deletion (frameshift)	9.36%
			CGACTGGCCAGG-----TGTGGG	5 bp deletion (frameshift)	6.99%
			CGACTGGCCAGGGCGCCTTGTGGG	1 bp insertion (frameshift)	5.24%
			CGACTGGCCAGGGC---TGTGGG	3 bp deletion (non-frameshift)	5.18%
			CGACTGGCCAGGG----TGTGGG	4 bp deletion (frameshift)	4.64%
			CGACTGGCCAGGGCGCCCTGTGGG	1 bp insertion (frameshift)	4.45%

Supplementary Table 2

Deep sequencing analysis of 8 off-target (OT) sites detected by WGS

Number	Chrom	Predicted OT pos	Indel pos	Distance (bp)	On-target seq	Predicted OT seq	Strand	Function	Mutation type	Validated by deep seq	Nucleotide repeats
1	chr15	34428733	34428916	182	CGACTGGCCAGGGCGCCTGTGGG	CGATAGGCCAGGGCGCCT-GCAG	-	intergenic	1-5 bp deletion<br>1 bp insertion	Yes	AAAAAAAAAAAAAAAA AAAAA
2	chr15	34574943	34575106	162	CGACTGGCCAGGGCGCCTGTGGG	CGATAGGCCAGGGCGCCT-GCAG	-	intergenic	NA	No	NA
3	chr15	34574943	34575120	176	CGACTGGCCAGGGCGCCTGTGGG	CGATAGGCCAGGGCGCCT-GCAG	-	intergenic	NA	No	NA
4	chr17	47703037	47702993	43	CGACTGGCCAGGGCGCCTGTGGG	CGAAGGGACAGGGGGTCTGTGAG	+	intronic	2 bp deletion	Yes	GGGGGGGGG
5	chr2	72911968	72912124	155	CGACTGGCCAGGGCGCCTGTGGG	TGAGTGTCTAGGGGGCCTGTAGG	+	intergenic	NA	No	NA
6	chr21	13246921	13246759	161	CGACTGGCCAGGGCGCCTGTGGG	CGTCTAGCCAGGGAGCATCTCAG	-	intergenic	4 bp insertion	Yes	TGTGTGTGTGTGTGTGT GTGTGTGTGTGTGTGTG
7	chr3	187139213	187139074	138	CGACTGGCCAGGGCGCCTGTGGG	GCAGAGGCCAGGGCGCCGGTAAG	-	intronic	NA	No	NA
8	chr9	131301652	131301813	160	CGACTGGCCAGGGCGCCTGTGGG	CGCCTGGCCCGGGAAGCTGTGGG	-	intronic	NA	No	NA

Supplementary Table 3

Deep sequencing analysis of top 29 off-target sites predicted by the Benchling CRISPR tool

Site	Edit Site	Target sequence	PAM	Mismatch	Validated by deep seq
On-target	chr2:+:241858825	CGACTGGCCAGGGCGCCTGT	GGG	0	NA
Off-target 1	chr12:+:590629	CCACTGCCCAGGGCGCCTGG	AAG	3	No
Off-target 2	chr3:+:135323926	CAACTGGCCAGGGCACCTAT	GAG	3	No
Off-target 3	chr1:+:1088609	CCACCGGCCAGGGCGCCTTT	AAG	3	No
Off-target 4	chr8:-:53878623	GGACTGGCCAGTGCGGCTGT	AGG	3	No
Off-target 5	chr15:-:78526890	AGACTGGCCAGGGAGTCTGT	GAG	3	No
Off-target 6	chr3:-:123344000	GGACTGGCCAAGGAGCCTGT	AGG	3	No
Off-target 7	chr21:+:43988998	CGTGTGGCCAGGGGGCCTGT	GAG	3	No
Off-target 8	chr11:-:62855728	TGCCTGGCCAGGGCGACTGG	CGG	4	No
Off-target 9	chr9:-:92171112	GGCCTGGCCAGGGCGGCTGG	GGG	4	No
Off-target 10	chr11:+:1153416	GGCCTGGCCAGGGCCCCTGC	TGG	4	No
Off-target 11	chr14:+:92376556	GGGCTGGCCAGGGCCCCTGA	GGG	4	No
Off-target 12	chr11:+:36455288	GGCCTGGCCAGGGAGCCTGG	GAG	4	No
Off-target 13	chr1:+:19454074	AGAGTGGCCAGGGCTCCTGG	AGG	4	No
Off-target 14	chr1:+:3982141	TGTCTGGCCAGGGTGCCTGC	TGG	4	No
Off-target 15	chr10:-:133474998	AGACAGGCCAGGGCACCTGC	AGG	4	No
Off-target 16	chr13:-:113104005	CTGCTGGCCAGGGCGGCTGC	AGG	4	No
Off-target 17	chr1:-:6103274	AGCCTGGCCAGGGCTCCTTT	GGG	4	No
Off-target 18	chr16:+:53503575	CAATTGGCCAGGGCGTCTGC	CAG	4	No
Off-target 19	chr5:-:758609	TGCCTGGCCATGGCGCCTGC	AGG	4	No
Off-target 20	chr9:+:109891067	CCCCTGGCCAGGGTGCCTGG	AGG	4	No
Off-target 21	chr8:+:11549226	GGACTGACCAGGGAGCCTGC	AGG	4	No
Off-target 22	chr2:+:239056117	CGCCAGGCCAGGGCGCCAGG	CAG	4	No
Off-target 23	chr2:-:229943631	CCACGGGCCAGGGCTCCTGA	CAG	4	No
Off-target 24	chr10:-:102415823	GGACTGGGCAGGGCACCTGG	AGG	4	No
Off-target 25	chr7:-:149765282	CCCCTGGCCAGCGCGCCTGG	CGG	4	No
Off-target 26	chr6:-:35078996	AGGCTGGCCAGGGCTCCAGT	GAG	4	No
Off-target 27	chr1:-:153642303	CCCCTGGCCAGGGCCCCTAT	GGG	4	No
Off-target 28	chr2:+:201929252	CCACAGGCCAGGGTGCCTGG	AAG	4	No
Off-target 29	chr5:+:1104177	TGACTGCCCAGGGCGTCTCT	GAG	4	No

Supplementary Table 4

Deep sequencing analysis of top 24 off-target sites identified by iGUIDE

Site	Gene ID	Edit Site	Target sequence	PAM	Mismatch	Abundance	Mutation type	Validated by deep seq
On-target	PDCD1*	chr2:+:241858824	CGACTGGCCAGGGCGCCTGT	GGG	0	616	NA	NA
Off-target 1	PHACTR1*	chr6:-:13230036	TTCATGGCCAGGGAGCCTGT	AGG	5	89	1 bp deletion	Yes
Off-target 2	FNBP1*~	chr9:-:129900386	CCTCTGGCAAGGGAGCCTGT	TGG	4	19	NA	No
Off-target 3	ADCY5*	chr3:-:123344000	GGACTGGCCAAGGAGCCTGT	AGG	3	15	NA	No
Off-target 4	CHTOP*	chr1:-:153642303	CCCCTGGCCAGGGCCCCTAT	GGG	4	12	NA	No
Off-target 5	LOC101928279	chr4:-:4758934	AGATCAGCCAGGGCACCTGT	AGG	5	8	NA	No
Off-target 6	PRKAG2*	chr7:+:151875244	GGCTGGCCCAGGGCCCCTGT	GGG	6	8	NA	No
Off-target 7	LOC101929406	chr1:-:29810368	CCTGGGCCCAGGGCGCCTGT	AGG	5	4	NA	No
Off-target 8	PKMYT1*	chr16:+:2980308	CGACTGGGCGGGGCGCGGGT	CCG	4	4	NA	No
Off-target 9	FLOT2*	chr17:-:28884726	GAATGGGCAAGGGCCCCTGT	GGG	6	3	NA	No
Off-target 10	CELF5*	chr19:+:3280103	CGACTGCCCAGGGCTGCCTG	TTG	6	3	NA	No
Off-target 11	LOC102724084*	chr16:-:80387613	TTCCTGGCCAGGGCATCTGT	AGG	5	2	NA	No
Off-target 12	ZC3H4*	chr19:-:47079954	CTCGTTGGCAGGGCGCCTGT	AGG	5	2	NA	No
Off-target 13	CFL1*	chr11:-:65858075	CGGCAGGCCACGGCGCCTGA	GGG	4	2	NA	No
Off-target 14	RPL3P4	chr14:-:98901704	TGGCTGGGCAGGGCCCCTGT	AGG	4	2	NA	No
Off-target 15	SERINC2*	chr1:-:31410498	CCACTGTCCTGGCCTCCTGC	CTG	6	2	NA	No
Off-target 16	CACUL1	chr10:+:118755373	GGACTGGCCAGAGTGATTGG	AGG	6	2	NA	No
Off-target 17	SAP130	chr2:-:128028289	GCCCTGGCCGGGTCGCCTGC	GGC	6	2	NA	No
Off-target 18	ZNF470	chr19:-:56567142	CAGCTCGCAGGAGCGCCTGT	CCG	6	2	NA	No
Off-target 19	SH2D6	chr2:+:85418426	CCACACGCCAGGACGCGTGT	AAG	5	2	NA	No
Off-target 20	ZNF580	chr19:-:55635144	CCACTGCCCTGGGGGCCACT	CTG	6	2	NA	No
Off-target 21	CCT8*	chr21:-:29073583	AGGCTGGCCATGGCGCTTCA	CGT	6	2	NA	No
Off-target 22	BCL3*~	chr19:-:44754665	CGCCTGGCCTGGGTCCCTCT	CGG	5	2	NA	No
Off-target 23	NECAP2*	chr1:-:16441615	CCATAGGCCAGGTTGTCTGT	ATG	6	2	NA	No
Off-target 24	MRPL46*	chr15:-:88467239	CGGATCCCCATGGCGCTTGT	TGG	6	2	NA	No

MESL, maximum edit site likelihood. Symbols after the gene name indicate: * the site is within the transcription unit of the gene, ~ the gene appears on the cancer-association list.

Supplementary Table 5

Infusion products of non-viral *PD1*-integrated CAR-T cells

Patient	Weight (kg)	CAR+ cell dose (/kg)	Total cell number	CAR+ (%)	Viability (%)	CD4/CD8 ratio	Culture days (after electroporation)
Patient-1	62	0.56×10^{6}	6.32×10^{8}	5.49	96.2	0.75	11
Patient-2	89	2.04×10^{6}	9.41×10^{8}	19.3	97.5	0.15	9
Patient-3	75	1.95×10^{6}	8.55×10^{8}	17.1	98.6	0.35	7
Patient-4	70	0.8×10^{6}	1.15×10^{9}	4.87	95.2	1.11	8
Patient-5	63	0.76×10^{6}	1.01×10^{8}	47.3	93.4	0.01	13
Patient-6	60	2.35×10^{6}	5.90×10^{8}	23.9	92.2	0.50	10
Patient-7	59	2.15×10^{6}	7.17×10^{8}	17.7	93.2	0.18	8
Patient-8	42	1.9×10^{6}	5.12×10^{8}	15.6	97.4	0.30	10

Supplementary Table 6

Prior lines of therapy for each patient

Patient	Number of prior lines of therapy	Prior therapies
Patient-1	5	R-CHOP<br>Sintilimab + ICE + R-Gemox + Chidamide<br>R-Gemox + Chidamide<br>Radiation therapy<br>Rituximab + Lenalidomide + Chidamide
Patient-2	4	R-COP<br>RCD<br>Lenalidomide<br>R-DA-EPOCH
Patient-3	3	R-CHOP<br>R2-CHOP<br>R-CHOPE
Patient-4	10	R2-CHOP<br>Rituximab + Lenalidomide<br>Rituximab<br>Rituximab+ Ibrutinib+ EPOCH<br>R-Hyper-CVADA<br>Rituximab+ Ibrutinib+ Hyper-CVADA<br>Rituximab+ Ibrutinib<br>ASCT<br>SMART<br>Brentuximab vedotin + Ibrutinib + CHOP
Patient-5	4	R-VDP<br>Hyper-CVADA<br>Hyper-CVADB<br>MTX + Asparaginase + DXM
Patient-6	4	R-CHOP<br>R-GDP<br>R-CHOP<br>R2-DA-EPOCH
Patient-7	1	R-CHOP
Patient-8	3	BR C1<br>BR C2<br>BR C3<br>BR C4<br>R2-CHOP<br>R-GDP

ASCT: autologous stem cell transplant; BR: bendamustine, rituximab; CHOP: cyclophosphamide, adriamycin, vincristine, prednisone; CHOPE: cyclophosphamide, adriamycin, vincristine, prednisone, etoposide; COP: cyclophosphamide, vincristine, prednisone; DA: dose adjustment; DXM: dexamethasone; EPOCH: etoposide, prednisone, vincristine, cyclophosphamide, doxorubicin; GDP: gemcitabine, dexamethasone, cisplatin; Hyper-CVADA: cyclophosphamide, vincristine, doxorubicin, dexamethasone; Hyper-CVADB: methetrexate, cytarabine; ICE: ifosfamide, carboplatin, etoposide; MTX: methotrexate; R: rituximab; R2: revlimid, rituximab; RCD: rituximab, cyclophosphamide, dexamethasone; R-GemOx: rituximab, gemcitabine, oxaliplatin; SMART: simultaneous modulated accelerated radiotherapy; VDP: vincristine, daunorubicin, prednisone.

Supplementary Table 7

Summary of adverse events in the study

AE category	Toxicity	All grades	Grade 1/2	Grade 3/4
CRS	/	4	4	0
Hematologic	Decrease in white blood cells	17	9	8
	Increase in white blood cells	1	1	0
	Decrease in lymphocytes	12	1	11
	Decrease in platelet	5	1	4
	Decrease in neutrophil	19	5	14
	Hypoalbuminemia	2	2	0
	Anaemia	5	1	4
	Low fibrinogen	1	1	0
Electrolyte	Hypocalcemia	4	4	0
	Alkaline phosphatase	1	1	0
Metabolic	Hypertriglyceridemia	4	4	0
Gastrointestinal	Abdominal distention	1	1	0
	Abdominal distention and nausea	1	1	0
	Nausea	1	1	0
	Dysphagia	1	1	0
	Throat discomfort	2	2	0
	Abdominal aching	2	2	0
Respiratory	Cough or with expectoration	2	2	0
	Thick breathing sounds	1	1	0
Neurologic	Headache	1	1	0
	Dizzy	1	1	0
Other	Fatigue	1	1	0
	Multiple lumps	1	1	0
	Small amount of urine	1	1	0
	Left leg swell	1	1	0
	Subcutaneous mass of left elbow	1	1	0
Total		93	52	41
