# Supplementary material for: Non-viral, specifically targeted CAR-T cells achieve high safety and efficacy in B-NHL
Source: Nature. 2022 Aug 31;609(7926):369–74. doi: 10.1038/s41586-022-05140-y (PMC9452296; doi:10.1038/s41586-022-05140-y)
Supplement: Supplementary file 2 — Reporting Summary [file 41586_2022_5140_MOESM2_ESM.pdf]

nature portfolio

Corresponding author(s): He Huang

Last updated by author(s): Jul 1, 2022

Reporting Summary

Nature Portfolio wishes to improve the reproducibility of the work that we publish. This form provides structure for consistency and transparency in reporting. For further information on Nature Portfolio policies, see our Editorial Policies and the Editorial Policy Checklist.

Statistics

For all statistical analyses, confirm that the following items are present in the figure legend, table legend, main text, or Methods section.

n/a	Confirmed	
☐	☒	The exact sample size (n) for each experimental group/condition, given as a discrete number and unit of measurement
☐	☒	A statement on whether measurements were taken from distinct samples or whether the same sample was measured repeatedly
☐	☒	The statistical test(s) used AND whether they are one- or two-sided<br>*Only common tests should be described solely by name; describe more complex techniques in the Methods section.*
☒	☐	A description of all covariates tested
☐	☒	A description of any assumptions or corrections, such as tests of normality and adjustment for multiple comparisons
☐	☒	A full description of the statistical parameters including central tendency (e.g. means) or other basic estimates (e.g. regression coefficient) AND variation (e.g. standard deviation) or associated estimates of uncertainty (e.g. confidence intervals)
☐	☒	For null hypothesis testing, the test statistic (e.g. F, t, r) with confidence intervals, effect sizes, degrees of freedom and P value noted<br>*Give P values as exact values whenever suitable.*
☒	☐	For Bayesian analysis, information on the choice of priors and Markov chain Monte Carlo settings
☒	☐	For hierarchical and complex designs, identification of the appropriate level for tests and full reporting of outcomes
☒	☐	Estimates of effect sizes (e.g. Cohen's d, Pearson's r), indicating how they were calculated

Our web collection on statistics for biologists contains articles on many of the points above.

Software and code

Policy information about availability of computer code

Data collection	Electronic Case Report Forms (eCRF) (v1.0) for clinical data. 4D-Nucleofector X Unit (Lonza) or GT Flow Transfection System (MaxCyte) for preparation of non-viral genome specific targeted (CAR) T cells. LSRFortessa (BD Biosciences) using FACSDiva software (v8.0.1) or DxFLEX Flow Cytometer (BECKMAN COULTER) using CytExpert software (v1.1.11.8) for flow cytometry analysis. FACSAria II (BD Biosciences) using FACSDiva software (v8.0.1) for FACS. NC-200 (ChemoMetec) or Countstar® BioMed (IM1200) for cell counting. SPECTROstar Nano (BMG LABTECH) using the software (v2.12) for OD detection. QuantStudio 3 Real-Time PCR System (Applied Biosystems) for qPCR. IVIS® Spectrum Imaging System (PerkinElmer) using Living Image® software (v4.5 or v4.7.3) for bioluminescence imaging. HiSeq X Ten platform (Illumina) for iGUIDE and deep sequencing. NovaSeq 6000 platform (Illumina) for WGS and scRNA-seq. Siemens Biograph16 PET/CT using Syngo VD30C SL18P60 software for PET/CT imaging. Automated immunostainer (Bond-III, Leica or Autostainer Link 48, Dako) using a Bond Polymer Refine Detection system for IHC staining.
Data analysis	BWA (v0.7.8-r455), SAMtools (v1.0), Sambamba (v0.4.7), picard-tools (v1.107), ANNOVAR (version 2017June8) for WGS data analysis. CellRanger (v3.1.0), Seurat package (v3.1.4), QuSAGE (v2.16.1), GSVA (v1.32.0), GSEA (http://broadinstitute.org/gsea) for scRNA-seq data analysis. Deep sequencing was analyzed using HiTOM analysis (Liu et al, SCIENCE CHINA Life Sciences, 2019). Indel percentage was analyzed using ICE v2 CRISPR Analysis Tool (Synthego, https://www.synthego.com/products/bioinformatics/crispr-analysis). Cas-Offinder (v2.4) (http://www.rgenome.net/cas-offinder/) or the Benchling CRISPR tool (https://www.benchling.com/) for predicting potential off-target sites. iGUIDE pipeline (https://github.com/cnobles/iGUIDE) for iGUIDE analysis. ImageJ (1.46r) for densitometry quantification. Flow cytometry data was analyzed using FlowJo (v10). GraphPad Prism (v7) was used to analyze other data.

For manuscripts utilizing custom algorithms or software that are central to the research but not yet described in published literature, software must be made available to editors and reviewers. We strongly encourage code deposition in a community repository (e.g. GitHub). See the Nature Portfolio guidelines for submitting code & software for further information.

March 2021

Data

Policy information about availability of data

All manuscripts must include a data availability statement. This statement should provide the following information, where applicable:

- Accession codes, unique identifiers, or web links for publicly available datasets
- A description of any restrictions on data availability
- For clinical datasets or third party data, please ensure that the statement adheres to our policy

ScRNA-seq data has been deposited in GEO database (GSE166352, GSE186596, GSE201035). WGS, iGUIDE and deep sequencing data have been deposited in SRA database (PRJNA774073, PRJNA772163, PRJNA772700, PRJNA772894, PRJNA772887, PRJNA772893).

Human research participants

Policy information about studies involving human research participants and Sex and Gender in Research.

Reporting on sex and gender	Male and female patients were enrolled in the clinical trial and reported in this study. Sex was not especially considered, as this was a consecutive cohort study referring to other reports. The disaggregated sex data and overall numbers were provided in Results.
Population characteristics	Eight patients were enrolled to receive the infusion of PD1-19bbz cells at a planned dose of 2×10^6 CAR T cells/kg. The population characteristics of them are shown in Table 1. Their ages range from 43 to 62 years old and sex is evenly distributed. Seven patients received at least three prior lines of therapy and one patient received one prior line of therapy. The details of prior therapies are provided in Supplementary table 3. Healthy human blood donors were male or female and mostly between 18 and 28 years old.
Recruitment	Patients were recruited through public recruitment advertisement which was approved by the First Affiliated Hospital, College of Medicine, Zhejiang University or through the recommendation from the hospital's doctors according to the medical history and results of routine tests. Eight patients were enrolled based on the inclusion and exclusion criteria in this clinical trial. The trial was approved by the institutional review board and all patients provided written informed consent in accordance with the Declaration of Helsinki before enrolment. Fresh PBMCs from healthy donors were provided by the First Affiliated Hospital, College of Medicine, Zhejiang University and Shanghai SAILY Biological Technology Co., Ltd. The recruitments of healthy human blood donors were approved by the Clinical Research Ethics Committee of the First Affiliated Hospital, College of Medicine, Zhejiang University and by Shanghai Zhaxin Traditional Chinese Ethics Committee and Western Medicine Hospital. All the donors signed the Informed Consent Form.
Ethics oversight	The clinical protocol was reviewed and approved by the Clinical Research Ethics Committee of the First Affliated Hospital, College of Medicine, Zhejiang University (2020IIT(85)).

Note that full information on the approval of the study protocol must also be provided in the manuscript.

Field-specific reporting

Please select the one below that is the best fit for your research. If you are not sure, read the appropriate sections before making your selection.

☒ Life sciences ☐ Behavioural & social sciences ☐ Ecological, evolutionary & environmental sciences

For a reference copy of the document with all sections, see nature.com/documents/nr-reporting-summary-flat.pdf

Life sciences study design

All studies must disclose on these points even when the disclosure is negative.

Sample size	This is an eight patient exploratory pilot study with a planned infusion dose of 2×10^6 CAR T cells/kg. If this study suggests the safety and feasibility of non-viral PD1-integrated CAR T cell therapy, a larger follow-on trial will be performed according to a standard 3+3 design for a phase I dose-escalation trial.
Data exclusions	In some analyses, the scRNA-seq data of the patient-3 samples were excluded due to an unreliable low CAR+ cell number. The data exclusions were described in the figure legends.
Replication	For the preclinical study, experiments were replicated in at least two independent healthy human donors unless otherwise noted.
Randomization	Randomization is not applicable as this is a phase I, open-label, single-arm study.
Blinding	There was no blinding as a phase I, open-label, single-arm clinical trial was performed.

Behavioural & social sciences study design

All studies must disclose on these points even when the disclosure is negative.

Study description	*Briefly describe the study type including whether data are quantitative, qualitative, or mixed-methods (e.g. qualitative cross-sectional, quantitative experimental, mixed-methods case study).*
Research sample	*State the research sample (e.g. Harvard university undergraduates, villagers in rural India) and provide relevant demographic information (e.g. age, sex) and indicate whether the sample is representative. Provide a rationale for the study sample chosen. For studies involving existing datasets, please describe the dataset and source.*
Sampling strategy	*Describe the sampling procedure (e.g. random, snowball, stratified, convenience). Describe the statistical methods that were used to predetermine sample size OR if no sample-size calculation was performed, describe how sample sizes were chosen and provide a rationale for why these sample sizes are sufficient. For qualitative data, please indicate whether data saturation was considered, and what criteria were used to decide that no further sampling was needed.*
Data collection	*Provide details about the data collection procedure, including the instruments or devices used to record the data (e.g. pen and paper, computer, eye tracker, video or audio equipment) whether anyone was present besides the participant(s) and the researcher, and whether the researcher was blind to experimental condition and/or the study hypothesis during data collection.*
Timing	*Indicate the start and stop dates of data collection. If there is a gap between collection periods, state the dates for each sample cohort.*
Data exclusions	*If no data were excluded from the analyses, state so OR if data were excluded, provide the exact number of exclusions and the rationale behind them, indicating whether exclusion criteria were pre-established.*
Non-participation	*State how many participants dropped out/declined participation and the reason(s) given OR provide response rate OR state that no participants dropped out/declined participation.*
Randomization	*If participants were not allocated into experimental groups, state so OR describe how participants were allocated to groups, and if allocation was not random, describe how covariates were controlled.*

Ecological, evolutionary & environmental sciences study design

All studies must disclose on these points even when the disclosure is negative.

Study description	*Briefly describe the study. For quantitative data include treatment factors and interactions, design structure (e.g. factorial, nested, hierarchical), nature and number of experimental units and replicates.*
Research sample	*Describe the research sample (e.g. a group of tagged Passer domesticus, all Stenocereus thurberi within Organ Pipe Cactus National Monument), and provide a rationale for the sample choice. When relevant, describe the organism taxa, source, sex, age range and any manipulations. State what population the sample is meant to represent when applicable. For studies involving existing datasets, describe the data and its source.*
Sampling strategy	*Note the sampling procedure. Describe the statistical methods that were used to predetermine sample size OR if no sample-size calculation was performed, describe how sample sizes were chosen and provide a rationale for why these sample sizes are sufficient.*
Data collection	*Describe the data collection procedure, including who recorded the data and how.*
Timing and spatial scale	*Indicate the start and stop dates of data collection, noting the frequency and periodicity of sampling and providing a rationale for these choices. If there is a gap between collection periods, state the dates for each sample cohort. Specify the spatial scale from which the data are taken*
Data exclusions	*If no data were excluded from the analyses, state so OR if data were excluded, describe the exclusions and the rationale behind them, indicating whether exclusion criteria were pre-established.*
Reproducibility	*Describe the measures taken to verify the reproducibility of experimental findings. For each experiment, note whether any attempts to repeat the experiment failed OR state that all attempts to repeat the experiment were successful.*
Randomization	*Describe how samples/organisms/participants were allocated into groups. If allocation was not random, describe how covariates were controlled. If this is not relevant to your study, explain why.*
Blinding	*Describe the extent of blinding used during data acquisition and analysis. If blinding was not possible, describe why OR explain why blinding was not relevant to your study.*

Did the study involve field work? ☐ Yes ☐ No

Field work, collection and transport

Field conditions	*Describe the study conditions for field work, providing relevant parameters (e.g. temperature, rainfall).*
Location	*State the location of the sampling or experiment, providing relevant parameters (e.g. latitude and longitude, elevation, water depth).*
Access & import/export	*Describe the efforts you have made to access habitats and to collect and import/export your samples in a responsible manner and in compliance with local, national and international laws, noting any permits that were obtained (give the name of the issuing authority, the date of issue, and any identifying information).*
Disturbance	*Describe any disturbance caused by the study and how it was minimized.*

Reporting for specific materials, systems and methods

We require information from authors about some types of materials, experimental systems and methods used in many studies. Here, indicate whether each material, system or method listed is relevant to your study. If you are not sure if a list item applies to your research, read the appropriate section before selecting a response.

Materials & experimental systems

n/a	Involved in the study	
☐	☒	Antibodies
☐	☒	Eukaryotic cell lines
☒	☐	Palaeontology and archaeology
☐	☒	Animals and other organisms
☐	☒	Clinical data
☒	☐	Dual use research of concern

Methods

n/a	Involved in the study	
☒	☐	ChIP-seq
☐	☒	Flow cytometry
☒	☐	MRI-based neuroimaging

Antibodies

Antibodies used

FITC anti-human CD3 (Biolegend, 300306, Clone HIT3a)
APC anti-human CD69 (Biolegend, 310910, Clone FN50)
APC anti-human CD137 (Biolegend, 309810, Clone 4B4-1)
APC anti-human CD25 (Biolegend, 356110, Clone M-A251)
APC anti-human PD1 (Biolegend, 329908, Clone EH12.2H7)
APC anti-human LAG3 (Biolegend, 369212, Clone 7H2C65)
APC anti-human TIM3 (Biolegend, 345012, Clone F38-2E2)
BV421 anti-human CD45RO (Biolegend, 304224, Clone UCHL1)
APC anti-human CD62L (Biolegend, 304809, Clone DREG-56)
APC anti-human CD3 (Biolegend, 300312, Clone HIT3A)
FITC anti-human CD19 (Biolegend, 302206, Clone HIB19)
FITC anti-human CD4 (Biolegend, 317408, Clone OKT4)
APC anti-human CD4 (Biolegend, 317416, Clone OKT4)
APC anti-human CD8 (Biolegend, 301014, Clone RPA-T8)
BV421 anti-human CD45 (Biolegend, 304032, Clone HI30)
PerCP-Cy™5.5 anti-human CD45 (BD Biosciences, 564105, Clone HI30)
PerCP-Cy™5.5 anti-human CD4 (BD Biosciences, 560650, Clone RPA-T4)
BV421 anti-human CD8 (BD Biosciences, 562428, Clone RPA-T8)
APC anti-human PD-L1 (ThermoFisher, 17-5983-41, Clone MIH1)
Anti-human CD19 (Biolynx, I1045, Clone BP6046)
Anti-human PD-L1 (Agilent, pharmDx, Clone 22C3)

Validation

All antibodies used in this study are commercially available. Antibody validations were performed by the suppliers and the information is provided on the website and product information datasheets. The certificate of analysis (CoA) was provided for the quality assurance of each antibody lot.

Eukaryotic cell lines

Policy information about cell lines and Sex and Gender in Research

Cell line source(s)

293T (CRL-3216™) and Nalm-6 (CRL-3273™) cells were purchased from ATCC. Raji cells were purchased from Cell Bank of Chinese Academy of Sciences.

Authentication

293T and Nalm-6 cells were directly purchased from ATCC with certification. 293T, Nalm-6 and Raji cells were authenticated

Authentication	by STR profiling by GENEWIZ Co., Ltd.
Mycoplasma contamination	All cell lines used were tested negative for mycoplasma contamination.
Commonly misidentified lines (See ICLAC register)	No commonly misidentified lines were used in the study.

Palaeontology and Archaeology

Specimen provenance	*Provide provenance information for specimens and describe permits that were obtained for the work (including the name of the issuing authority, the date of issue, and any identifying information). Permits should encompass collection and, where applicable, export.*
Specimen deposition	*Indicate where the specimens have been deposited to permit free access by other researchers.*
Dating methods	*If new dates are provided, describe how they were obtained (e.g. collection, storage, sample pretreatment and measurement), where they were obtained (i.e. lab name), the calibration program and the protocol for quality assurance OR state that no new dates are provided.*

☐ Tick this box to confirm that the raw and calibrated dates are available in the paper or in Supplementary Information.

Ethics oversight	*Identify the organization(s) that approved or provided guidance on the study protocol, OR state that no ethical approval or guidance was required and explain why not.*

Note that full information on the approval of the study protocol must also be provided in the manuscript.

Animals and other research organisms

Policy information about studies involving animals; ARRIVE guidelines recommended for reporting animal research, and Sex and Gender in Research

Laboratory animals	For the experiment comparing LV-19bbz and AAVS1-19bbz groups at a high infusion dose, we used 6- to 8-week-old B-NDG (NOD.CB17-PrkdcscidIl2rgtm1/Bcgen) male mice (Biocytogen). For other experiments, we used 6- to 9-week-old NCG (NOD/ShiLtJGpt-Prkdcem26Cd52Il2rgem26Cd22/Gpt) female mice (GemPharmatech).
Wild animals	N/A
Reporting on sex	Only one sex was used in the individual mouse experiments according to the general knowledge in the field. The disaggregated sex data and overall numbers were provided in Results.
Field-collected samples	N/A
Ethics oversight	All animal experiments were conducted in compliance with “Guide for the Care and Use of Laboratory Animals (2011)” issued by the National Research Council (USA), “Laboratory Animal Administration Regulation (2017)” issued by the National Science and Technology Committee (China), and the laboratory animal administration regulations (Shanghai, Jiangsu). The care and the use of animals were reviewed and approved by the Institutional Animal Care and Use Committee (IACUC) of the East China Normal University Center for Animal Research or InnoStar Bio-tech Nantong Co., Ltd.

Note that full information on the approval of the study protocol must also be provided in the manuscript.

Clinical data

Policy information about clinical studies

All manuscripts should comply with the ICMJE guidelines for publication of clinical research and a completed CONSORT checklist must be included with all submissions.

Clinical trial registration	ClinicalTrials.gov (NCT04213469)
Study protocol	The study protocol was described in Methods.
Data collection	Clinflash EDC, an electronic data capture system, was used in this study. All data collection was assured to be complete and accurate in the electronic case report forms according to the protocol. All data collected from April 2020.
Outcomes	The primary endpoints of this study were safety and feasibility. Secondary endpoint was efficacy. Exploration objectives included the assessments of in vivo persistence and peak blood levels of PD1-19bbz cells.

Dual use research of concern

Policy information about dual use research of concern

Hazards

Could the accidental, deliberate or reckless misuse of agents or technologies generated in the work, or the application of information presented in the manuscript, pose a threat to:

No	Yes	
☐	☐	Public health
☐	☐	National security
☐	☐	Crops and/or livestock
☐	☐	Ecosystems
☐	☐	Any other significant area

Experiments of concern

Does the work involve any of these experiments of concern:

No	Yes	
☐	☐	Demonstrate how to render a vaccine ineffective
☐	☐	Confer resistance to therapeutically useful antibiotics or antiviral agents
☐	☐	Enhance the virulence of a pathogen or render a nonpathogen virulent
☐	☐	Increase transmissibility of a pathogen
☐	☐	Alter the host range of a pathogen
☐	☐	Enable evasion of diagnostic/detection modalities
☐	☐	Enable the weaponization of a biological agent or toxin
☐	☐	Any other potentially harmful combination of experiments and agents

ChIP-seq

Data deposition

☐ Confirm that both raw and final processed data have been deposited in a public database such as GEO.

☐ Confirm that you have deposited or provided access to graph files (e.g. BED files) for the called peaks.

Data access links<br>*May remain private before publication.*	*For "Initial submission" or "Revised version" documents, provide reviewer access links. For your "Final submission" document, provide a link to the deposited data.*
Files in database submission	*Provide a list of all files available in the database submission.*
Genome browser session<br>(e.g. UCSC)	*Provide a link to an anonymized genome browser session for "Initial submission" and "Revised version" documents only, to enable peer review. Write "no longer applicable" for "Final submission" documents.*

Methodology

Replicates	*Describe the experimental replicates, specifying number, type and replicate agreement.*
Sequencing depth	*Describe the sequencing depth for each experiment, providing the total number of reads, uniquely mapped reads, length of reads and whether they were paired- or single-end.*
Antibodies	*Describe the antibodies used for the ChIP-seq experiments; as applicable, provide supplier name, catalog number, clone name, and lot number.*
Peak calling parameters	*Specify the command line program and parameters used for read mapping and peak calling, including the ChIP, control and index files used.*
Data quality	*Describe the methods used to ensure data quality in full detail, including how many peaks are at FDR 5% and above 5-fold enrichment.*
Software	*Describe the software used to collect and analyze the ChIP-seq data. For custom code that has been deposited into a community repository, provide accession details.*

Flow Cytometry

Plots

Confirm that:

☒ The axis labels state the marker and fluorochrome used (e.g. CD4-FITC).

☒ The axis scales are clearly visible. Include numbers along axes only for bottom left plot of group (a 'group' is an analysis of identical markers).

☒ All plots are contour plots with outliers or pseudocolor plots.

☒ A numerical value for number of cells or percentage (with statistics) is provided.

Methodology

Sample preparation	Cell culture and the procedures of sample preparation and collection are described in Methods. Cells were pre-washed and incubated with antibodies for 30 minutes on ice. After washing twice, samples were analyzed or sorted by flow cytometry.
Instrument	LSRFortessa (BD Biosciences) or DxFLEX Flow Cytometer (BECKMAN COULTER) for flow cytometry analysis. FACSAria II (BD Biosciences) for FACS.
Software	FlowJo (version 10) was used for flow cytometry data analysis.
Cell population abundance	For Sanger sequencing of AAVS1-19bbz and PD1-19bbz samples and genotyping of PD1-19bbz cells, CAR+ cells were sorted by FACS in advance.
Gating strategy	FSC-A/SSC-A plots were used to determine cell population gates. FSC-A/FSC-H plots were then used to determine singlet gates. The 7-AAD viability dye was used to distinguish live cells from dead cells. Additional gating was performed as described in the figure legends for individual experiments.

☒ Tick this box to confirm that a figure exemplifying the gating strategy is provided in the Supplementary Information.

Magnetic resonance imaging

Experimental design

Design type	*Indicate task or resting state; event-related or block design.*
Design specifications	*Specify the number of blocks, trials or experimental units per session and/or subject, and specify the length of each trial or block (if trials are blocked) and interval between trials.*
Behavioral performance measures	*State number and/or type of variables recorded (e.g. correct button press, response time) and what statistics were used to establish that the subjects were performing the task as expected (e.g. mean, range, and/or standard deviation across subjects).*

Acquisition

Imaging type(s)	*Specify: functional, structural, diffusion, perfusion.*
Field strength	*Specify in Tesla*
Sequence & imaging parameters	*Specify the pulse sequence type (gradient echo, spin echo, etc.), imaging type (EPI, spiral, etc.), field of view, matrix size, slice thickness, orientation and TE/TR/flip angle.*
Area of acquisition	*State whether a whole brain scan was used OR define the area of acquisition, describing how the region was determined.*

Diffusion MRI ☐ Used ☐ Not used

Preprocessing

Preprocessing software	*Provide detail on software version and revision number and on specific parameters (model/functions, brain extraction, segmentation, smoothing kernel size, etc.).*
Normalization	*If data were normalized/standardized, describe the approach(es): specify linear or non-linear and define image types used for transformation OR indicate that data were not normalized and explain rationale for lack of normalization.*
Normalization template	*Describe the template used for normalization/transformation, specifying subject space or group standardized space (e.g. original Talairach, MNI305, ICBM152) OR indicate that the data were not normalized.*
Noise and artifact removal	*Describe your procedure(s) for artifact and structured noise removal, specifying motion parameters, tissue signals and physiological signals (heart rate, respiration).*

Volume censoring | *Define your software and/or method and criteria for volume censoring, and state the extent of such censoring.*

Statistical modeling & inference

Model type and settings | *Specify type (mass univariate, multivariate, RSA, predictive, etc.) and describe essential details of the model at the first and second levels (e.g. fixed, random or mixed effects; drift or auto-correlation).*

Effect(s) tested | *Define precise effect in terms of the task or stimulus conditions instead of psychological concepts and indicate whether ANOVA or factorial designs were used.*

Specify type of analysis: ☐ Whole brain ☐ ROI-based ☐ Both

Statistic type for inference
(See Eklund et al. 2016) | *Specify voxel-wise or cluster-wise and report all relevant parameters for cluster-wise methods.*

Correction | *Describe the type of correction and how it is obtained for multiple comparisons (e.g. FWE, FDR, permutation or Monte Carlo).*

Models & analysis

n/a	Involved in the study
☐	☐ Functional and/or effective connectivity
☐	☐ Graph analysis
☐	☐ Multivariate modeling or predictive analysis

Functional and/or effective connectivity | *Report the measures of dependence used and the model details (e.g. Pearson correlation, partial correlation, mutual information).*

Graph analysis | *Report the dependent variable and connectivity measure, specifying weighted graph or binarized graph, subject- or group-level, and the global and/or node summaries used (e.g. clustering coefficient, efficiency, etc.).*

Multivariate modeling and predictive analysis | *Specify independent variables, features extraction and dimension reduction, model, training and evaluation metrics.*
